# Genomic characterization of WRKY transcription factors related to secoiridoid biosynthesis in *Gentiana macrophylla*

**DOI:** 10.1186/s12870-024-04727-z

**Published:** 2024-01-23

**Authors:** Yangyang Yin, Huanhuan Fu, Fakai Mi, Ye Yang, Yaomin Wang, Zhe Li, Yihan He, Zhenggang Yue

**Affiliations:** 1 State Key Laboratory of Research & Development of Characteristic Qin Medicine Resources (Cultivation), Coconstruction Collaborative Innovation Center for Chinese Medicinal Resources Industrialization By Shaanxi & Education Ministry, Shaanxi Innovative Drug Research Center, School of Pharmacy, Shaanxi University of Chinese Medicine, Xianyang, 712046 People’s Republic of China; 2 College of Life Science, Qinghai Normal University, Xining, 810016 People’s Republic of China

**Keywords:** *Gentiana macrophylla*, WRKY transcription factors, Secoiridoid biosynthesis, Correlation analysis, Methyl jasmonate treatment

## Abstract

*Gentiana macrophylla* is one of Chinese herbal medicines in which 4 kinds of iridoids or secoiridoids, such as loganic acid, sweroside, swertiamarin, and gentiopicroside, are identified as the dominant medicinal secondary metabolites. WRKY, as a large family of transcription factors (TFs), plays an important role in the synthesis of secondary metabolites in plants. Therefore, WRKY genes involved in the biosynthesis of secoiridoids in *G. macrophylla* were systematically studied. First, a comprehensive genome-wide analysis was performed, and 42 *GmWRKY* genes were identified, which were unevenly distributed in 12 chromosomes. Accordingly, gene structure, collinearity, sequence alignment, phylogenetic, conserved motif and promoter analyses were performed, and the GmWRKY proteins were divided into three subfamilies based on phylogenetic and multiple sequence alignment analyses. Moreover, the enzyme-encoding genes of the secoiridoid biosynthesis pathway and their promoters were then analysed, and the contents of the four secoiridoids were determined in different tissues. Accordingly, correlation analysis was performed using Pearson′s correlation coefficient to construct WRKY gene-enzyme-encoding genes and WRKY gene–metabolite networks. Meanwhile, *G. macrophylla* seedlings were treated with methyl jasmonate (MeJA) to detect the dynamic change trend of *GmWRKYs*, biosynthetic genes, and medicinal ingredient accumulation. Thus, a total of 12 *GmWRKYs* were identified to be involved in the biosynthesis of secoiridoids, of which 8 (*GmWRKY1*, *6*, *12*, *17*, *33*, *34*, *38* and *39*) were found to regulate the synthesis of gentiopicroside, and 4 (*GmWRKY7*, *14*, *26* and *41*) were found to regulate the synthesis of loganic acid. Taken together, this study systematically identified WRKY transcription factors related to the biosynthesis of secoiridoids in *G. macrophylla*, which could be used as a cue for further investigation of *WRKY* gene functions in secondary metabolite accumulation.

**Supplementary Information:**

The online version contains supplementary material available at 10.1186/s12870-024-04727-z.

## Introduction

*Gentiana macrophylla* Pall, a member of the herbaceous plant in the genus *Gentiana* of the Gentianaceae family, is found mainly in the Loess Plateau and the eastern part of the Qinghai–Tibet Plateau of China [1]. As one of the famous traditional herbs, the roots of *G. macrophylla*, called “Qin-jiao” in Chinese, have been used for the therapy of allergic inflammation [2] and antirheumatic [3], anti-inflammatory and pain treatments for rheumatic diseases [4]. In addition, iridoid or secoiridoid glycosides have been identified as the dominant medicinal secondary metabolites, of which loganic acid, seweroside, swertiamarin and gentiopicroside are used as the key characteristic biomarkers for the quality control of *G. macrophylla* [5, 6]. It is well known that iridoids are synthesized via two pathways: the cytosolic mevalonic acid (MVA) pathway [7] and the plastidial 2C-methyl-D-erythritol 4-phosphate (MEP) pathway [8] in plants. However, the biosynthesis of secoiridoids involved in *G. macrophylla* is still unclear.

Recent investigations have shown that transcription factors (TFs) are powerful tools to improve the yield and quality of active ingredients by regulating the expression of enzyme-encoding genes involved in biosynthetic pathways [9]. In plants, many TFs (such as MYB, bHLH, bZIP, MADS-box, WRKY and WDR) have been found to participate in the regulation of secondary metabolite biosynthesis [10], among which WRKY, as one of the largest members in higher plants, is also the most studied TF in the regulation of plant function [11]. WRKY proteins have a highly conserved domain containing an almost invariant WRKYGQK sequence at the N-terminus followed by a C2H2 (CX4-5CX22-23HX1H) or C2HC (CX7CX23HXC) zinc-finger motif [12]. Based on both the number of WRKY domains and the features of their zinc-finger motifs, the WRKY proteins can be categorized into three distinct groups. Generally, group I has two WRKY domains and a C2H2 zinc-finger motif; group II has one WRKY domain and a C2H2 zinc-finger motif; and group III has one WRKY domain and a different C2HC zinc-finger motif. Among them, the group II proteins are further divided into 5 subgroups a ~ e, based on additional conserved structural motifs outside the WRKY domain [13, 14].

The WRKY domain can activate or inhibit the transcription of downstream target genes by binding to their DNA element known as the W-box [15, 16], which then plays important roles in responding to plant abiotic stresses [17], participating in plant growth processes, participating in MAPK-mediated signal transduction processes [18], promoting plants to respond to exogenous hormones [19, 20], and ultimately regulating the accumulation of secondary metabolites [21]. For instance, overexpression of *CrWRKY1* in hairy roots of *Catharanthus roseus* upregulated several key TIA pathway genes. *CrWRKY1* combined with the W-box element of the tryptophan decarboxylase (TDC) promoter increased *TDC* activity, thereby promoting the synthesis of terpenoid indole alkaloids [22]. Overexpression of *OpWRKY2* in *Ophiorrhiza pumila* led to a more than threefold increase in camptothecin levels. *OpWRKY2* directly binds and activates the central camptothecin pathway gene *OpTDC* to affect the biosynthesis of camptothecin [23]. Transient overexpression of *TcWRKY33* in leaves of *Taxus chinensis* resulted in increased Taxol and 10-deacetylbaccatin accumulation by 1.20 and 2.16 times compared with the control, respectively [23]. Treating grape berries with ABA decreased the transcription level of *VviWRKY40* but increased the transcription level of *VviGT14*, a monoterpene β-D-glucosyltransferase, indicating that *VviWRKY40* is located downstream of the ABA signal transduction network to regulate monoterpenoid glycosylation [24]. All of the above studies indicate that WRKY genes play an important role in regulating the biosynthesis of secondary metabolites in plants. Since the first WRKY gene was identified in sweet potato [25], an increasing number of WRKY family members have been identified, depending on the development of available whole-genome sequences. For example, 50 *EcWRKYs* in *California Poppy* [26], 69 *SmWRKYs* in *Salvia milliorrhiza* [27], 137 *PgWRKYs* in *Panax ginseng* [28], and 39 *CasWRKYs* in *Cannabis sativa* [29] have been reported. However, *GmWRKYs* regulating the biosynthesis of secoiridoid glycosides in *G. macrophylla* are still unresolved.

In this study, the genome-wide identification and characterization of 42 *GmWRKYs* in *G. macrophylla* was performed using available genomic information. A comprehensive analysis of the *GmWRKYs,* including protein characterization, chromosome location, collinearity analysis, sequence alignment, phylogenetic tree, gene structure, conserved motif and promoter *cis*-element prediction, was performed. Moreover, based on the function of WRKY transcription factors in regulating enzyme-encoding gene expression to accumulate metabolite production, the enzyme-encoding genes in the biosynthetic pathway of iridoids were mined, and the promoter *cis-*elements of the enzyme-encoding genes were predicted. Based on the transcriptome data, the expression levels of *GmWRKYs* and biosynthetic enzyme-encoding genes were analysed. Using HPLC, the contents of the secondary metabolites in different organs (seed, root, leaf, flower and stem) of *G. macrophylla* were determined. Thus, the correlation between *GmWRKYs* and metabolic products, as well as the correlation between *GmWRKYs* and enzyme-encoding genes, were established to elucidate the relationship between *GmWRKYs* and medicinal ingredients. A previous study reported that treatment with methyl jasmonate (MeJA), an effective elicitor, increased both the expression of WRKY genes and the production of gentiopicroside in *G. macrophylla*, suggesting that WRKY genes could respond to MeJA elicitation, regulate enzyme-encoding genes in metabolic pathways, and then enhance the production of gentiopicroside [30]. We speculated that some *GmWRKYs* may act as a bridge between the MeJA signaling pathway and gentiopicroside in *G. macrophylla*. Based on this hypothesis, the seedlings of *G. macrophylla* were treated with MeJA, and then, the expression of *GmWRKY*s and secoiridoid biosynthetic enzyme-encoding genes, as well as the accumulation of secondary metabolites, were investigated. In brief, the aim of the present study was to identify WRKY transcription factors in *G. macrophylla* and reveal their molecular mechanism involved in secoiridoid biosynthesis*.*

## Materials and methods

### Plant materials and treatments

*G. macrophylla* plant samples grown for 3 years were collected from Fengxian County, Baoji City, Shaanxi Province, P. R. China (33.92′ N, 106.52′ E) and identified by Prof. Wei Wang (Shaanxi University of Chinese Medicine). A voucher specimen (No. GM-20191020) has been deposited in the Herbarium of Shaanxi University of Chinese Medicine (herbarium code: SNTCM). Whole roots, stems (axillary buds and leaves removed), leaves (top-leaves) and whole flowers were collected in mid-June, and immature seeds were collected in late July. All materials were dried in the shade for metabolite extraction, with 9 plants each time.

*G. macrophylla* seedlings were cultivated under the same conditions in the laboratory. At the four-leaf stage (grown for 3 months), the seedling materials were sprayed using 200 μM MeJA, collected and flash-frozen in liquid nitrogen stored at -80 °C at 0, 6, 12 and 24 h for RNA extraction, 6 plants each time; collected and dried in the shade on 0, 3 and 6 days for the content determination of the secondary metabolites, 30 plants each time. The normal seedlings were collected each time and set up as the blank control.

### Identification and protein property analysis of the *WRKY *gene family in *G. macrophylla*

The genome of *G. macrophylla* used in this study at NCBI under accession number PRJNA924980. The raw data of the RNA-seq analysis used in this study were submitted to the Sequence Read Archive (SRA) at NCBI under accession number SRR8438983-SRR8438986. The nucleotide and amino acid sequences of 72 *AtWRKYs* were downloaded from the Arabidopsis Information Resource (TAIR; available online: http://www.Arabidopsis.org/).

By mining the annotation information of the *G. macrophylla* transcriptome and alignment screening with known sequences, we initially identified 74 sequences annotated as WRKY transcription factors. First, the amino acid sequences of the 74 putative *WRKY* genes were isolated from the genome database of *G. macrophylla*. Next, all of these sequences were searched for sequence similarity by NCBI-Blast alignment, sequence family features were compared with SMART (http://smart.embl-heideberg.de/), and sequence alignment was performed using DNAMAN software. Finally, 42 *GmWRKYs* with complete WRKY domains were identified in *G. macrophylla*.

ExPASy (http://ExPASy.org/) [31] was used to investigate the length, molecular weight (MW), isoelectric points (PIs), instability index, and aliphatic index of WRKY proteins in *G. macrophylla*. Plant-mPLoc (http://www.csbio.sjtu.edu.cn/bioinf/plant-multi/) [32] was used to estimate the subcellular locations of WRKY proteins in the plant.

### Phylogenetic, gene structure and conserved motif analyses

The amino acid sequences in the WRKY domain of *G. macrophylla* and *A. thaliana* were aligned by MEGA 7.0 software [33] using the ClustalX program, and the phylogenetic analysis was constructed using the neighbour-joining method with 1,000 bootstrap replicates. Based on the genome annotation files of *G. macrophylla*, the structure of *GmWRKYs* was visualized using TBtools software [34]. Motifs were identified by the MEME 5.5.1 online program (http://meme-suite.org/tools/meme) [35] with the following parameters: number of repetitions, default; maximum number of motifs, 10; and optimum width of each motif, between 20 and 50 residues.

### Chromosomal locations, collinearity analysis and *cis*-acting regulatory prediction

TBtools software was used to locate all *GmWRKYs* on the chromosome of *G. macrophylla* and conduct collinearity analysis.

The 2 kb promoter sequences of *GmWRKY*s and the related enzyme-encoding genes, upstream of the translation initiation site, were obtained from the *G. macrophylla* genome. PlantCARE (http://bioinformatics.psb.ugent.be/webtools/plantcare/html/) [36] and PLACE (https://www.dna.affrc.go.jp/PLACE/?Action=newplace) were used to analyse these gene promoters and identify their *cis*-acting regulatory.

### Identification of enzyme-encoding genes related to the biosynthesis of secoiridoids

The biosynthesis pathway of the secoiridoids in *G. macrophylla* was constructed based on the KEGG pathway database [37] and related literature reports on the secoiridoid biosynthetic pathway [38]. Accordingly, the transcriptome annotation files of the roots, stems, leaves, flowers, and seeds were analysed to retrieve the names of the enzyme-encoding genes in the biosynthesis pathway and screen their numbers and sequences. Meanwhile, a local database was built with BioEidt software to align homologous genes of the metabolic pathway genes with tBlastX. Next, all enzyme-encoding gene sequences were searched for sequence similarity by NCBI-Blast alignment, and the same class of sequence alignment was performed using DNAMAN software to remove the repeat sequences. Finally, 84 enzyme-encoding genes were screened from the roots, stems, leaves, flowers and seeds according to the FPKM values (> 5.0).

### Content determination of the secondary metabolites

Source of standard products: loganic acid (purity: 99.12%), swertiamarin (purity: 99.51%), gentiopicroside (purity: 99.90%) and sweroside (purity: 99.16%) were all purchased from Shanghai Yuanye Bio-Technology Co., Ltd.

Each dried sample (50 mg) was extracted in 1 mL methanol under ultrasonic waves 3 times for 40 min each time and centrifuged for 5 min at 12,000 rpm, and the supernatant was collected and filtered through a 0.22 μm filter (Jiangsu Green Union Science Instrument Co., Ltd., Taizhou, China). Repeat 3 times. The contents of four main secondary metabolites, loganic acid, sweroside, swertiamarin and gentiopicroside, were determined via LC-20ADXR HPLC system Shimadzu (Japan) with 4.6 mm × 250 mm, C18 5 μm particles (Welch, USA); UV detector, 254 nm; column temperature, 30 °C; flow rate, 0.8 mL/min, using gradient solvent system ACN (Welch, USA) /H_2_O as the mobile phase (0—13 min, ACN 11%, 13—16 min, ACN 10%; 16—30 min, ACN 12%). Each injection volume was set at 20 μL.

The standard curve was constructed from the content of metabolite (*X*, mg/mL) and peak area (*Y*). The linear regression equation was *Y* = 9,313,169.91* x* + 52,842.65 (linear range 0.004375 mg/mL—1.12 mg/mL) for loganic acid, *Y* = 19,491,752.98 *x* + 131,241.49 (linear range, 0.004414063 mg/mL—1.13 mg/mL) for sweroside, *Y* = 10,348,224.18 *x* + 124,625.728 (linear range, 0.008007813 mg/mL—2.05 mg/mL) for gentiopicroside, and *Y* = 40,500,359.78* x* + 332,031.12 (linear range, 0.004921875 mg/mL—1.26 mg/mL) for swertiamarin. Each group was repeated 3 times. Excel 2019 was used for data processing, and GraphPad Prism 9.5 software was used for drawing and *t* test for significance.

### Association analysis of *WRKY* genes with enzyme-encoding genes and secondary metabolites in *G. macrophylla*

To obtain candidate *GmWRKYs* for the regulation of secoiridoid biosynthesis, according to the transcriptome data, one Pearson correlation analysis was performed between the expression of *GmWRKYs* and the genes involved in secoiridoid biosynthesis in different tissues, with a threshold of correlation coefficient 0.75, *p* < 0.05; the other Pearson correlation analysis was performed between the expression of *GmWRKYs* and the contents of secondary metabolites (gentiopicroside, sweroside, swertiamarin and loganic acid), with a threshold of correlation coefficient 0.75, *p* < 0.05.

### Gene expression analysis by RT–qPCR

Total RNA was isolated using the polysaccharide polyphenol plant total RNA extraction kit (Bioteke, Beijing). The concentration and purity of RNA were detected by Nanodrop (Thermo Fisher, USA), and its integrity was detected by 1% agarose gel electrophoresis (Solarbio, Beijing). Reverse transcription was then performed according to the manufacturer’s instructions using PrimeScript™ IV 1st strand cDNA Synthesis Mix (TaKaRa, Dalian) and stored at -20 °C until use. RT–qPCR (qTOWER 2.0, Analytik Jena, Germany) was performed under the following conditions: 95 °C for 30 s; 95 °C for 5 s and 60 °C for 20 s, 40 cycles. Relative expression of the genes was calculated by the 2^−∆∆Ct^ method. The *SAND1* gene was tested as an internal reference [39]. The primer information is shown in Additional file 13: Table S9. The results are represented by their means ± SDs. GraphPad Prism 9.5 software was used for drawing, and a *t* test was used for significance.

### Statistical analysis

All data are expressed as the mean ± SD of three independent biological replicates. Statistical analysis was performed using GraphPad Prism 9.5 software. A* t* test was used for statistical analysis, followed by an unpaired t test. *P* < 0.05 was considered to indicate a statistically significant difference.

## Results

### Identification and protein characterization of *WRKY* in *G. macrophylla*

To extensively identify the potential *WRKY* genes, according to the annotated genome data, a total of 74 putative WRKY gene family members were obtained using “WRKY” as the search term*.* The redundant sequences and the incomplete sequences without the zinc finger structure were expurgated, according to the results of NCBI-Blast alignment and SMART database comparison. Thus, a total of 42 *WRKY* genes were eventually identified and named *GmWRKY1*—*GmWRKY42* (Additional file 5: Table S1). The sequence analysis results showed that the protein length of these GmWRKYs ranged from 207 (GmWRKY24) to 707 (GmWRKY40) amino acids (aa), the protein relative molecular mass varied from 23.38 (GmWRKY24) to 77.37 (GmWRKY40) kDa, and the protein PI was distributed in a wide range from acidic 5.32 (GmWRKY2 and GmWRKY20) to very basic 9.9 (GmWRKY19). The instability index results showed that most GmWRKY proteins were greater than 40, belonging to the unstable proteins, except GmWRKY35 (38.39). The aliphatic index results showed that the proteins ranged from 35.69 (GmWRKY13) to 75.17 (GmWRKY14). The GRAVY scores of GmWRKY proteins were all below 0, indicating their hydrophilic characteristics. Furthermore, all 42 proteins were located in the nucleus.

### Classification of the *WRKY* proteins and phylogenetic analysis in *G. macrophylla*

Sequence analysis revealed that all GmWRKY proteins have the conserved “WRKYGQK” motif, except GmWRKY9, which possesses “WRKYGKK” (Additional file 1: Fig. S1). To understand the evolutionary relationship of GmWRKY proteins, a total of 42 GmWRKYs in *G. macrophylla* and 68 AtWRKYs in *A. thaliana* were used to build a phylogenetic tree constructed by the NJ method (Additional file 2: Fig. S2). Thus, 42 GmWRKY proteins were divided into three main groups, similar to other plants, in which group I included 12 GmWRKY proteins (GmWRKY 3, 8, 10, 12, 16, 17, 21, 25, 27, 38, 40, and 41); group II included 25 GmWRKY proteins, further refined into five subgroups: IIa (GmWRKY1), IIb (GmWRKY 5, 15, 32 and 39), IIc (GmWRKY 4, 9, 11, 13, 18, 24, 28, 34, 35 and 37), IId (GmWRKY 19, 22, 23, 26, 29, 31 and 36), and IIe (GmWRKY6, 33 and 42); and group III included 5 GmWRKY proteins (GmWRKY2, 7, 14, 20 and 30).

### Gene structure and conserved motifs of *WRKY* genes in *G. macrophylla*

The conserved motif and intron/exon distribution were visualized using TBtools software to clarify the structural characteristics of *GmWRKYs*. The gene structure map (Additional file 3: Fig. S3B) showed that among the 42 identified *GmWRKYs*, 2 contained one exon belonging to group IId; 1 contained two exons belonging to group IIc; 17 had three exons belonging mainly to groups II and III; 9 had four exons belonging only to group I; and 6 had five exons and 7 had six exons belonging mainly to group I, suggesting that *GmWRKY* members from the same WRKY group tended to share a similar gene structure in terms of intron/exon organization. The gene conserved motif map showed that a total of 10 motif patterns were obtained (Additional file 3: Fig. S3C and Additional file 6: Table S2). All family members of *GmWRKYs* contained motifs 1 and 2, whereas motifs 3, 7 and 10 were found only in groups I, IIb and IId, respectively; motifs 4 and 6 were mainly present in group I; motif 9 was found in groups I and IIc; motif 5 was mainly found in groups IId and IIe; and motif 8 was present in most genes in groups I and IId (Additional file 3: Fig. S3A). As expected, most of the *GmWRKYs* observed in the same group or subgroup usually shared highly similar motif compositions, further validating the phylogenetic relationship of *GmWRKYs* and suggesting that they might have a similar regulatory function in the same group.

### Chromosome locations and collinearity analysis of *WRKY* genes in *G. macrophylla*

**Fig. 1:**
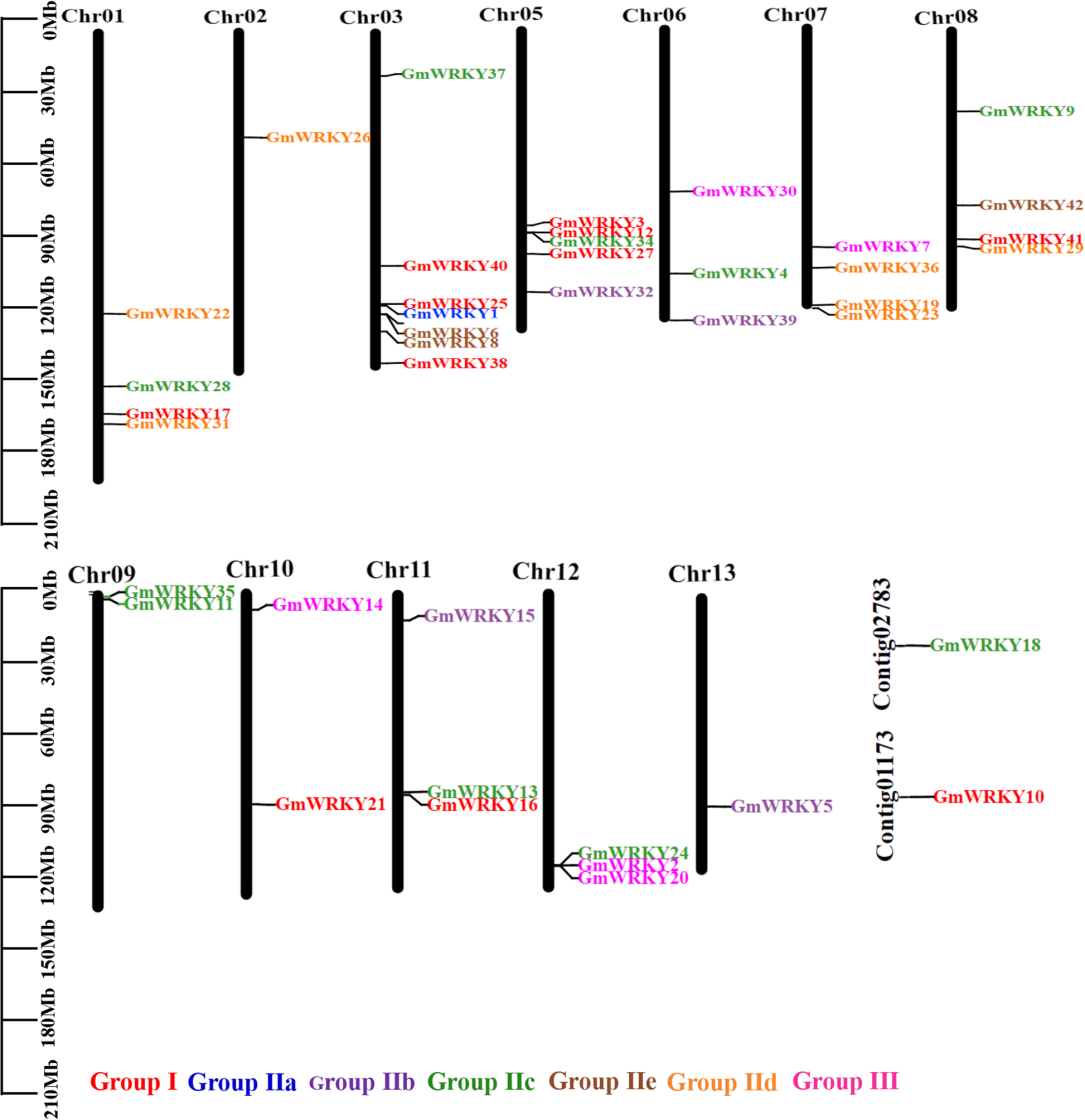
Distribution of *GmWRKYs* across the chromosomes of *G. macrophylla*. The chromosome numbers are shown at the top of each chromosome (black bars). The location of each *WRKY* gene is indicated by a line. Groups and subgroups are distinguished by different colors (groups: I-red; IIa-blue; IIb-purple; IIc-green; IId-yellow; IIe-brown; III-pink)

**Fig. 2:**
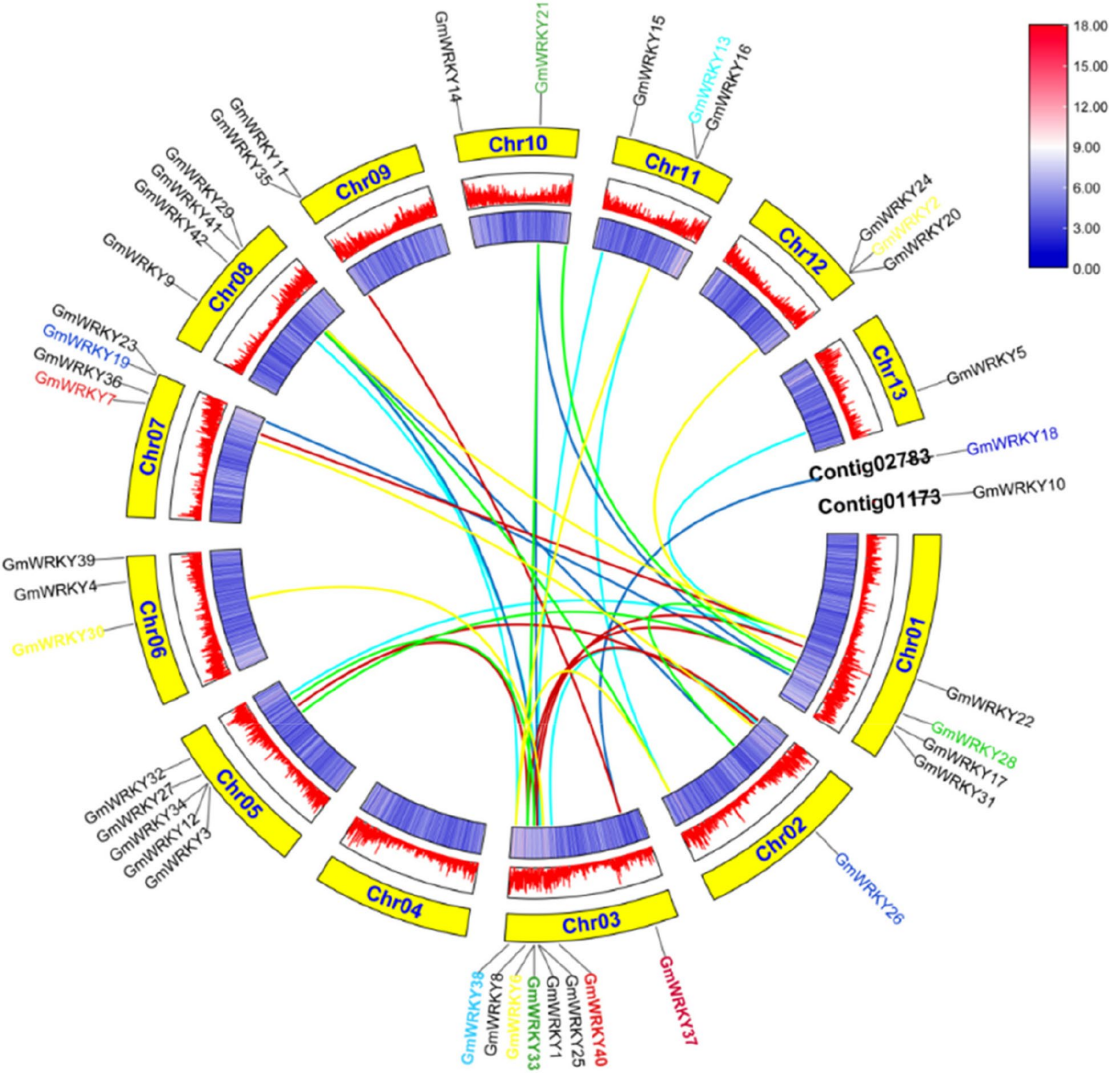
Synteny analyses of *GmWRKYs* in *G. macrophylla.* The yellow blocks indicate the parts of *G. macrophylla* chromosomes. The coloured lines indicate duplicated *GmWRKY* gene pairs. The scale bar on the top-right corner shows the different colors to represent the gene distribution density values. Low and high values are indicated in blue and red, respectively. Darker blue indicates less gene distribution, while darker red indicates more gene distribution

To determine the genomic distribution of *WRKY* genes, the physical map positions of 42 *GmWRKYs* were identified by TBtools based on annotation information of *G. macrophylla* (Fig. 1 and Additional file 7: Table S3), in which 40 *GmWRKYs* were distributed across 12 chromosomes, except *GmWRKY10* and *GmWRKY18*. Among these chromosomes, Chr3 had the most *WRKY* genes (8 genes, 19.5% of the total), followed by Chr5 (5 genes, 11.9% of the total), while Chr1, 7 and 8 contained 4 genes; Chr6, 11 and 12 contained 3 genes; Chr9 and 10 contained 2 genes; and Chr2 and 13 contained only one. Subsequently, TBtools was used to investigate their duplication events to illustrate the expansion patterns of *GmWRKYs* (Fig. 2). The results showed that 14 duplication pairs were identified, concentrated in groups I, IIc, IId, IIe and III. Two or more homologous genes within a 200 kb range on the same chromosome are defined as tandem repeat events [40]. In this study, two sets of tandem repeat events were found in 42 WRKYs, namely *GmWRKY33* and *GmWRKY6* located on chromosome 3, and *GmWRKY24*, *GmWRKY2*, and *GmWRKY20* located on chromosome (Additional file 8: Table S4).

### *Cis*-Acting regulatory elements in the promoter region of *WRKY* genes in *G. macrophylla*

**Fig. 3:**
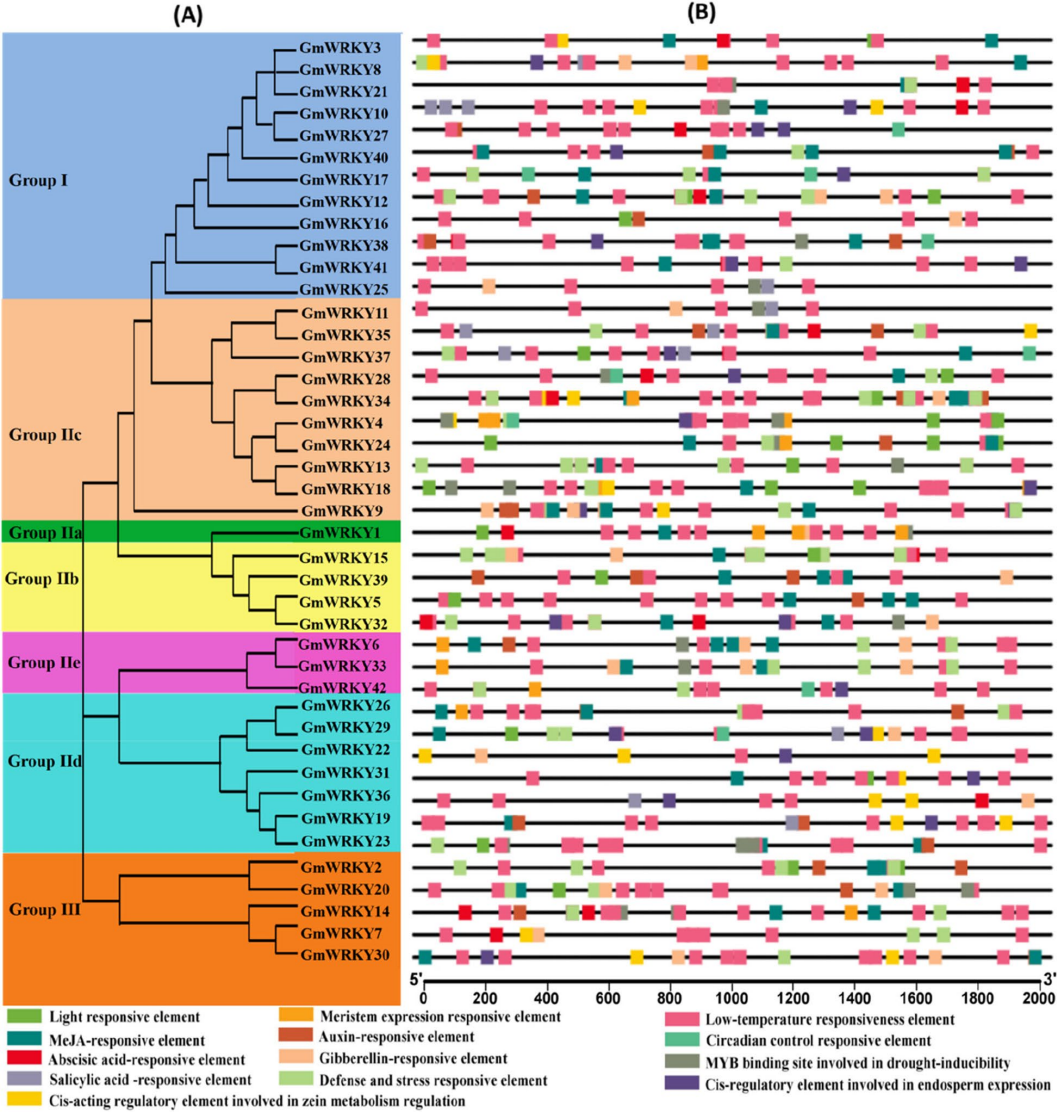
Prediction of *cis*-regulatory elements in the promoter regions of *GmWRKYs*. (A) Phylogenetic tree of GmWRKY proteins. (B) The distribution of *cis*-regulatory elements within each *GmWRKY* promoter

The *cis*-acting elements in the 2.0 kb upstream promoter region of all *GmWRKYs* were predicted and analysed by PlantCARE (Fig. 3). The results showed that many types of *cis*-acting elements were present. Among them, light responsive elements were found in the promoter region of 42 *GmWRK*Ys, accounting for the largest proportion, followed by MeJA-responsive elements (34 *GmWRKYs*). Abscisic acid- and auxin-responsive elements were found in 30 and 20 promoters of *GmWRKYs*, respectively. Gibberellin- and salicylic acid-responsive elements were found in the promoter regions of 20 and 15 genes, respectively. In addition, some elements related to abiotic stresses were found, such as low temperature (19 *GmWRKYs*), defense (17 *GmWRKYs*), and drought (19 *GmWRKYs*). Moreover, some elements were found to regulate plant growth and development, such as zein metabolism regulation, circadian control, endosperm expression and meristem expression.

### Expression analysis of *WRKY* genes in different tissues of *G. macrophylla*

**Fig. 4:**
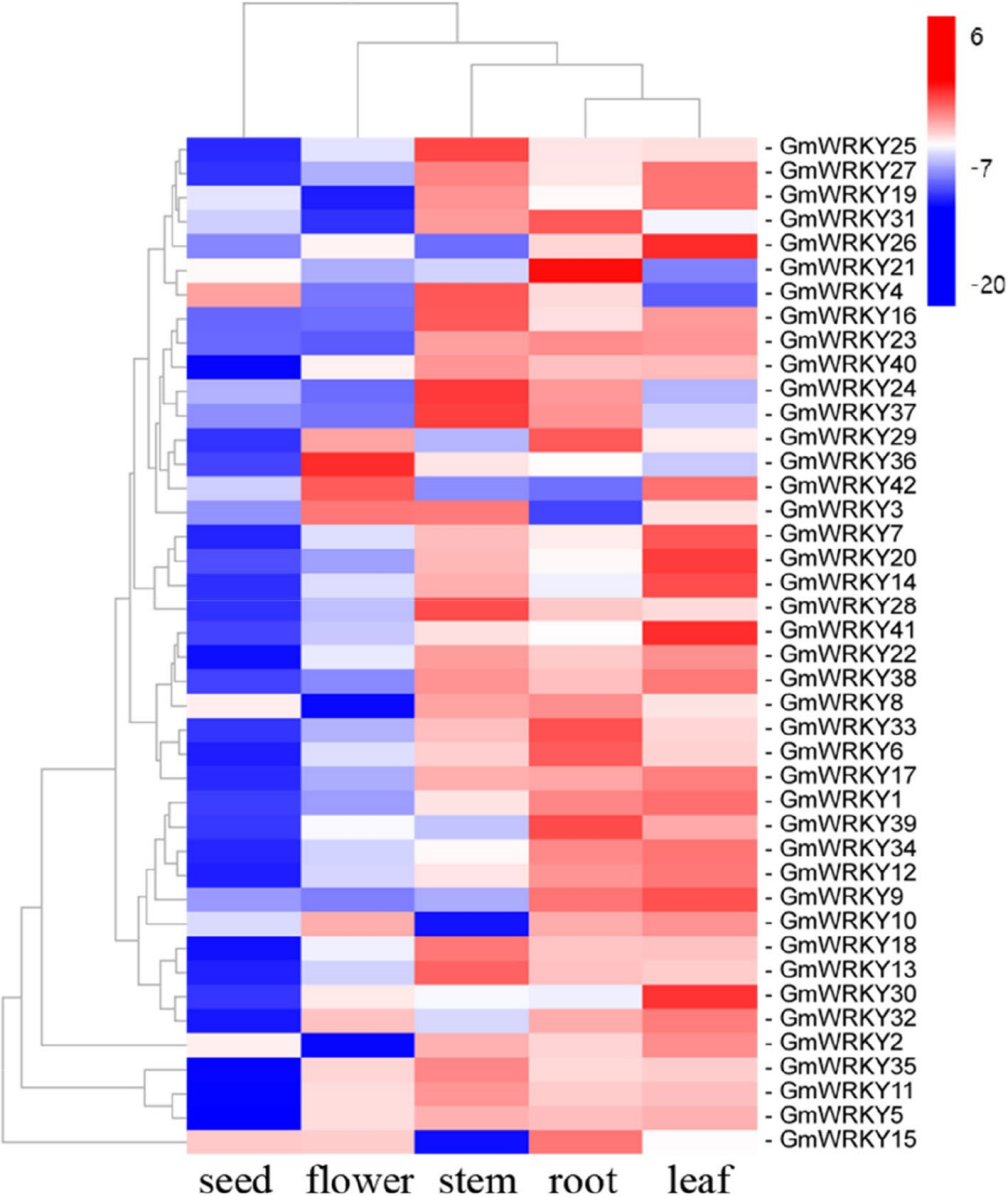
Heatmap representing the expression profiles of *GmWRKYs* in leaves, roots, stems, flowers and seeds. Different color blocks represent the normalized gene expression levels (log2 (FPKM)) of all genes in different tissues. Within each row, low and high values are indicated in light blue and red, respectively. The scale represents the signal intensity of FPKM values

It is well known that tissue-specific expression patterns of genes can provide invaluable information for understanding the biological function of genes. According to the transcriptome data, the expression profiles of *GmWRKYs* were generated, and the results showed that all 42 *GmWRKYs* were ubiquitously expressed in different tissues but with different expression patterns in the roots, stems, leaves, flowers and seeds (Fig. 4). Among them, 7 (*GmWRKY6*, *15*, *21*, *29*, *31*, *33* and *39*), 6 (*GmWRKY7*, *14*, *20*, *26*, *30* and *41*) and 7 (*GmWRKY4*, *13*, *16*, *24*, *25*, *28* and *37*) showed the highest levels expression in the roots, leaves and stems, respectively. In addition, some *GmWRKYs* were expressed in two tissues at high levels, such as *GmWRKY1*, *GmWRKY9*, *GmWRKY12* and *GmWRKY34* in roots and leaves, *GmWRKY19* and *GmWRKY27* in roots and stems, *GmWRKY42* in roots and flowers, and *GmWRKY3* in stems and flowers. The results indicated that these genes might be important for plant growth and organ development in *G. macrophylla*.

### Identification of the enzyme-encoding genes involved in secoiridoid biosynthesis

**Fig. 5:**
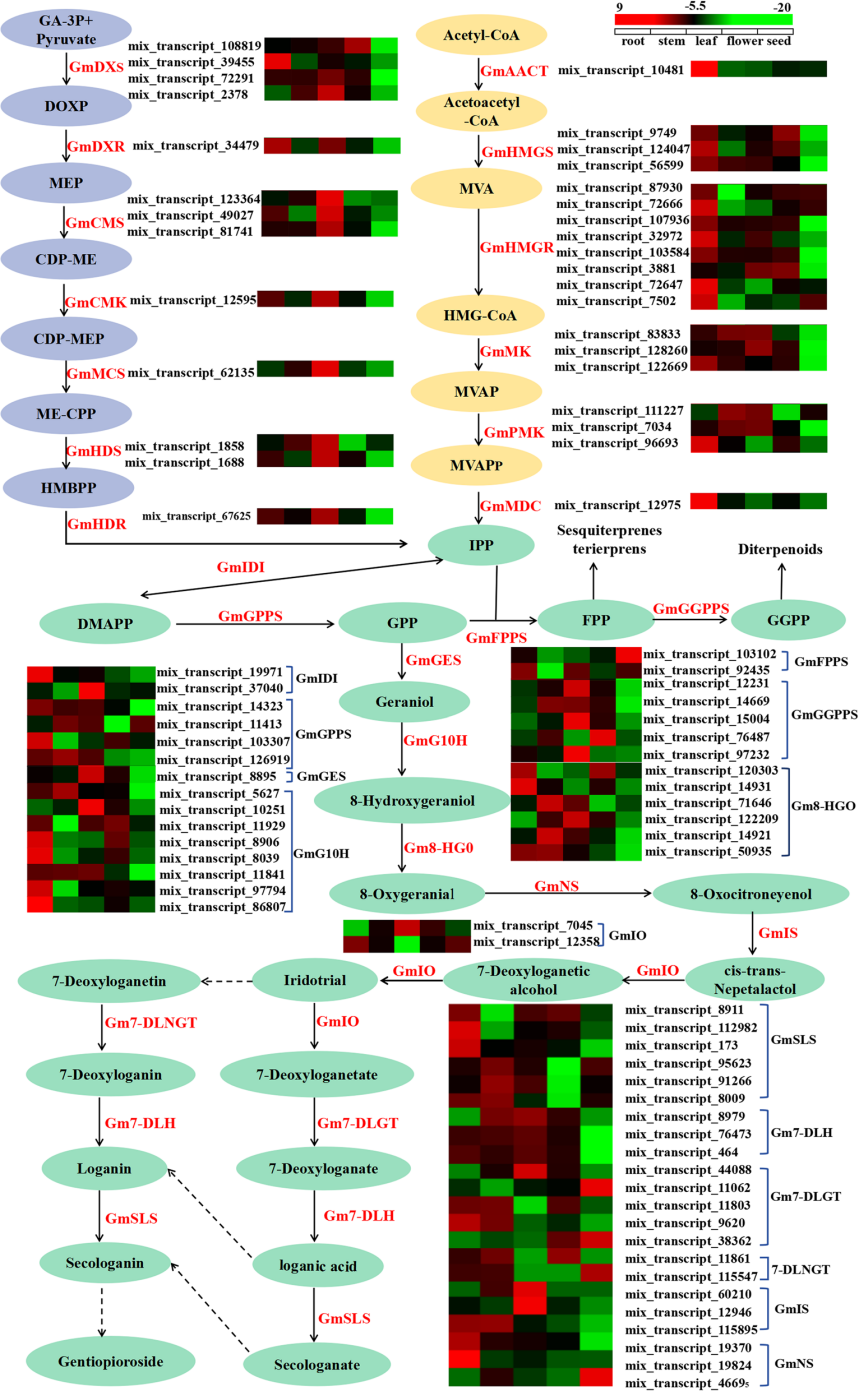
Analysis of gene expression involved in the secoiridoid biosynthesis of *G. macrophylla*. Different color blocks represent the normalized gene expression levels (log2 (FPKM)) of all genes in different tissues. The yellow ellipse represents the MVA pathway, the light blue ellipse represents the MEP pathway, and the green ellipse represents the characteristic secoiridoid pathway. GmAACT, acetoacetyl-CoA thiolase; GmHMGS, 3-hydroxy-3-methylglutaryl-CoA synthase; GmHMGR, 3-hydroxy-3-methylglutaryl-CoA reductase; GmMK, mevalonate kinase; GmPMK, phosphomevalonate kinase; GmMDC, mevalonate-5-pyrophosphate decarboxylase; GmDXS, deoxyd-xylulose-5-phosphate synthase; GmDXR, deoxy-D-xylulose-5-phosphate reductoisomerase; GmCMS, 4-(cytidine 5’-diphospho)-2-C-methyl-D-erythritol synthase; GmCMK, 4-(cytidine 5’-diphospho)-2-C-methyl-D-erythritol kinase; GmMCS, 2-C-methyld-erythritol2,4-cyclodiphosphate synthase; GmHDS, 4-hydroxy-3-methylbut-2-enyl diphosphate synthase; GmHDR, 4-hydroxy-3-methylbut-2-enyl diphosphate reductase; GmIDI, isopentenyl diphosphate isomerase; GmGPPS, geranyl diphosphate synthase; GmFPPS, farnesyl diphosphate synthase; GmGGPPS, geranylgeranyl diphosphate synthases; GmGES, geraniol synthase; GmG10H geraniol 10-hydroxylase; Gm8-HGO, 8-hydroxy-geraniol oxidoreductase; GmIS, iridoid synthase; GmNS, nepetalactol synthase; GmIO, iridoid oxidase; Gm7-DLGT, 7-deoxyloganetic acid glucosyltransferase; Gm7-DLNGT, 7-deoxyloganetic glucosyltransferase; Gm7-DLH, 7-deoxyloganate 7-hydroxylase; and GmSLS, secologanin synthase; were recorded as the key enzymes in the pathway

In simple terms, the secoiridoid biosynthesis pathway in plants can be divided into three main parts: the initial MEP and MVA pathways and the characteristic secoiridoid pathway [38]. Accordingly, the putative secoiridoid biosynthetic pathway in *G. macrophylla* was reconstructed based on the KEGG database. Thus, a total of 84 enzyme-encoding genes were classified into 27 enzyme categories related to the pathway (Fig. 5 and Additional file 9: Table S5). Among them, 19, 13 and 52 enzyme-encoding genes encoding 6, 7 and 14 enzymes were involved in the MVA, MEP and secoiridoid pathways, respectively. Furthermore, the enzyme-encoding genes of the MVA pathway were mainly present with the highest expression in roots but the lowest expression in seeds; the enzyme-encoding genes of the MEP pathway were mainly present with the highest expression in leaves but the lowest expression in seeds; and the enzyme-encoding genes of the characteristic secoiridoid pathway were mainly present with high expression in roots, stems and leaves but low expression in flowers and seeds.

### Correlation analysis of *WRKY* genes and the enzyme-encoding genes involved in secoiridoid biosynthesis in *G. macrophylla*

**Fig. 6:**
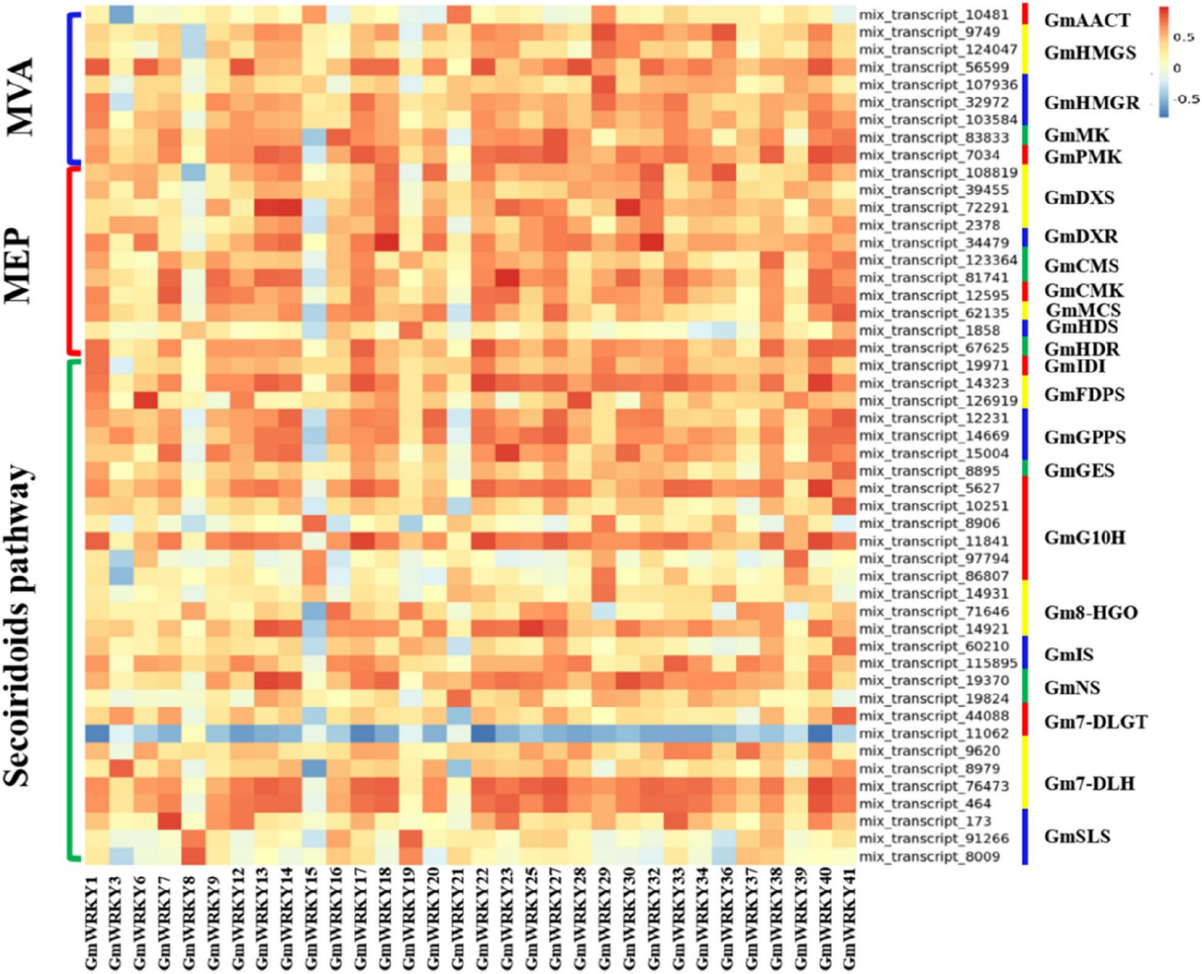
Pearson correlation analysis of *GmWRKYs* and the enzyme-encoding genes involved in secoiridoid biosynthesis in *G. macrophylla.* The scale bar on the top-right corner shows the different colors to represent the correlation coefficient values. The deeper the red is, the stronger the positive correlation. The deeper the blue is, the stronger the negative correlation

Pearson correlation analysis was performed to identify the *WRKY* genes involved in the regulation of secoiridoid biosynthesis in *G. macrophylla* (Fig. 6 and Additional file 10: Table S6). The results showed that 32 *GmWRKYs* were significantly correlated with 49 enzyme-encoding genes (encoding 23 enzymes). Among them, significant correlations were observed between 5 enzymes of the MVA pathway (*GmAACT*, *GmHMGS*, *GmHMGR*, *GmMK* and *GmPMK*) and 21 *GmWRKYs* (*GmWRKY1*, *6*, *12*, *13*, *14*, *17*, *18*, *21*, *22*, *23*, *25*, *27*, *28*, *29*, *30*, *32*, *33*, *36*, *38*, *40* and *41*), between 7 enzymes of the MEP pathway (*GmDXS*, *GmDXR*, *GmCMS*, *GmCMK*, *GmMCS*, *GmHDS* and *GmHDR*) and 21 *GmWRKYs* (*GmWRKY1*, *6*, *7*, *9*, *13*, *14*, *17*, *18*, *19*, *20*, *22*, *23*, *27*, *28*, *30*, *32*, *33*, *36*, *38*, *40* and *41*), between 11 enzymes of the characteristic secoiridoids pathway (*GmIDI*, *GmFDPS*, *GmGGPS*, *GmGES*, *GmG10H*, *Gm8-HGO*, *GmIS*, *GmNS*, *Gm7-DLGT*, *Gm7-DLH* and *GmSLS*) and 31 *GmWRKYs* (*GmWRKY1*, 3, *6*, *7*, 8, *9*, 12, *13*, *14*, 15, 16, *17*, *18*, *19*, *20*, 21, *22*, *23*, 25, *27*, *28*, *29*, *30*, *32*, *33*, *34*, 37, *38*, 39, *40* and *41*).

### Promoter analysis of the enzyme-encoding genes involved in secoiridoid biosynthesis in *G. macrophylla*

The mechanism of WRKY transcription factor regulation in activating or inhibiting gene expression involves binding to the W-box element in the target gene promoter. According to the above results that 49 enzyme-encoding genes were significantly related to 32 *GmWRKYs* in secoiridoid biosynthesis of *G. macrophylla*, these enzyme-encoding gene promoters were analysed (Additional file 11: Table S7). The results showed that eight types of W-boxes (ELRECOREPCRP1, WBBOXPCWRKY1, WBOXATNPR1, WBOXHVISO1, WBOXNTERF3, WBOXNTCHN48, TGACGTVMAMY and WRKY71OS) were present in the promoters. Each enzyme-encoding gene contained at least two kinds of W-boxes, one of which was WRKY71OS. Each enzyme-encoding gene contained at least two kinds of W-boxes, one of which was WRKY71OS. These results implied that 32 *GmWRKYs* might directly bind to W-box elements in regulating the expression of the key enzyme-encoding genes in *G. macrophylla* and thereby affecting secoiridoid biosynthesis.

### Correlation analysis of *WRKY *gene expression with the content of secondary metabolites in *G. macrophylla*

**Fig. 7:**
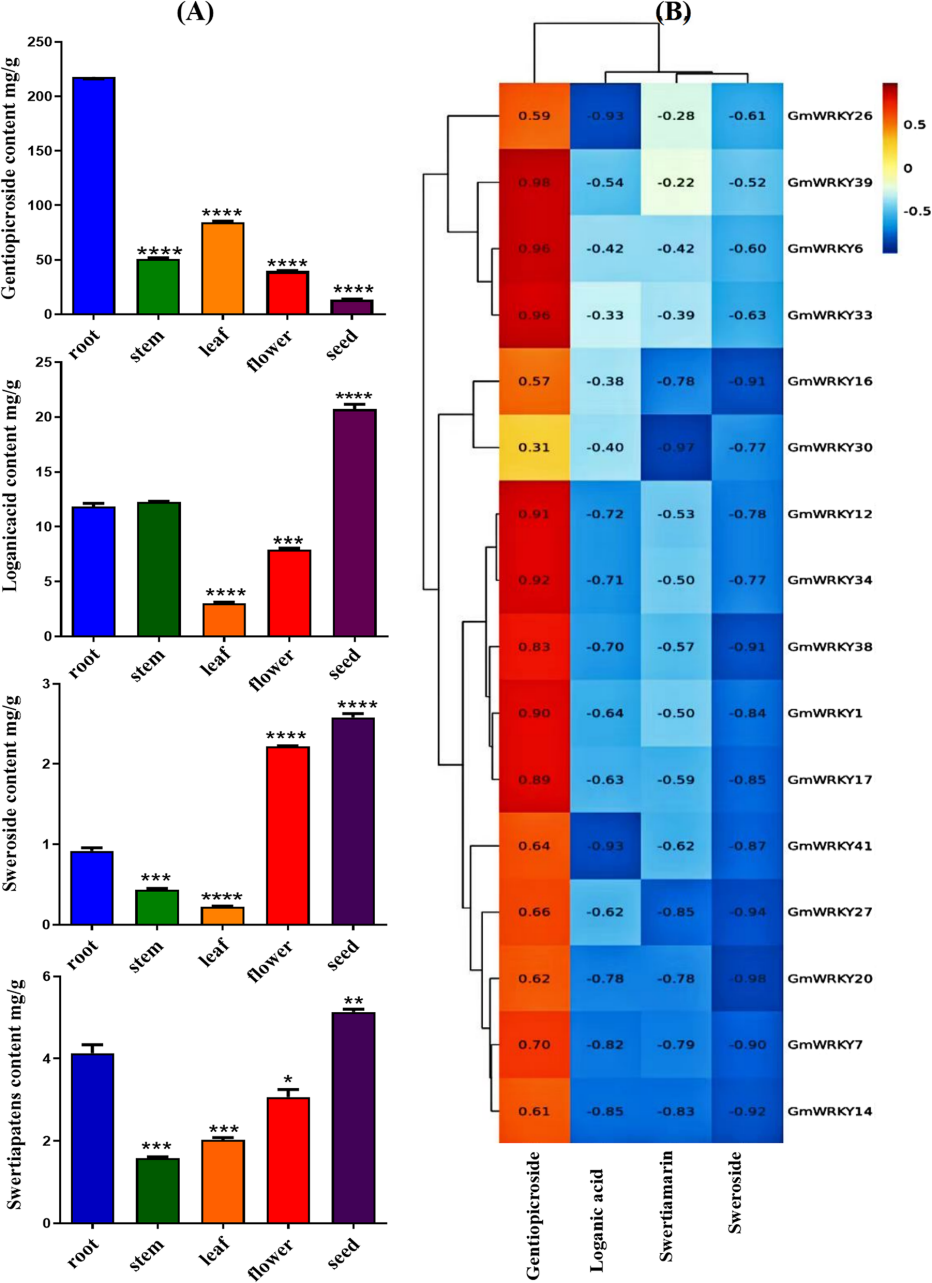
Correlation analysis of *WRKY* expression with the content of secondary metabolites in *G. macrophylla.* (A) Content determination of four representative components (loganic acid, swertiamarin, gentiopicroside and sweroside) in different tissues. The roots were used as a blank control. The asterisks (*) represent significant differences (*, *P* < 0.05; **, *P* < 0.01; ***, *P* < 0.001; and ****, *P* < 0.0001). (B) Correlation analysis of *GmWRKY* expression with the content of the four components

HPLC was used to determine the contents of four representative components, namely, loganic acid, swertiamarin, sweroside, and gentiopicroside, in different tissues. The results (Fig. 7A and Additional file 12: Table S8) showed that gentiopicroside was present at the highest level in roots at 217.98 ± 2.87 mg/g (dry weight), while loganic acid, swertiamarin, and sweroside were present at the highest levels in seeds at 20.69 ± 0.63 mg/g, 5.13 ± 0.10 mg/g and 2.58 ± 0.06 mg/g, respectively. The correlation of the expression of 32 *GmWRKY*s with the contents of these components was then analysed. The results showed (Fig. 7B) that 16 *GmWRKYs* displayed a significant correlation with the components, of which 8 (*GmWRKY1*, *6*, *12*, *17*, *33*, *34*, *38* and *39*) presented a positive correlation with gentiopicroside, while 4 (*GmWRKY7*, *14*, *26* and *41*), 3 (*GmWRKY14*, *27* and *30*) and 8 (*GmWRKY1*, *7*, *14*, *16*, *20*, *27*, *38* and *41*) were negatively correlated with loganic acid, swertiamarin and sweroside, respectively.

### Analysis of *WRKY* gene expression, biosynthetic enzyme-encoding gene expression, and component content determination in MeJA-treated *G. macrophylla* seedlings

**Fig. 8:**
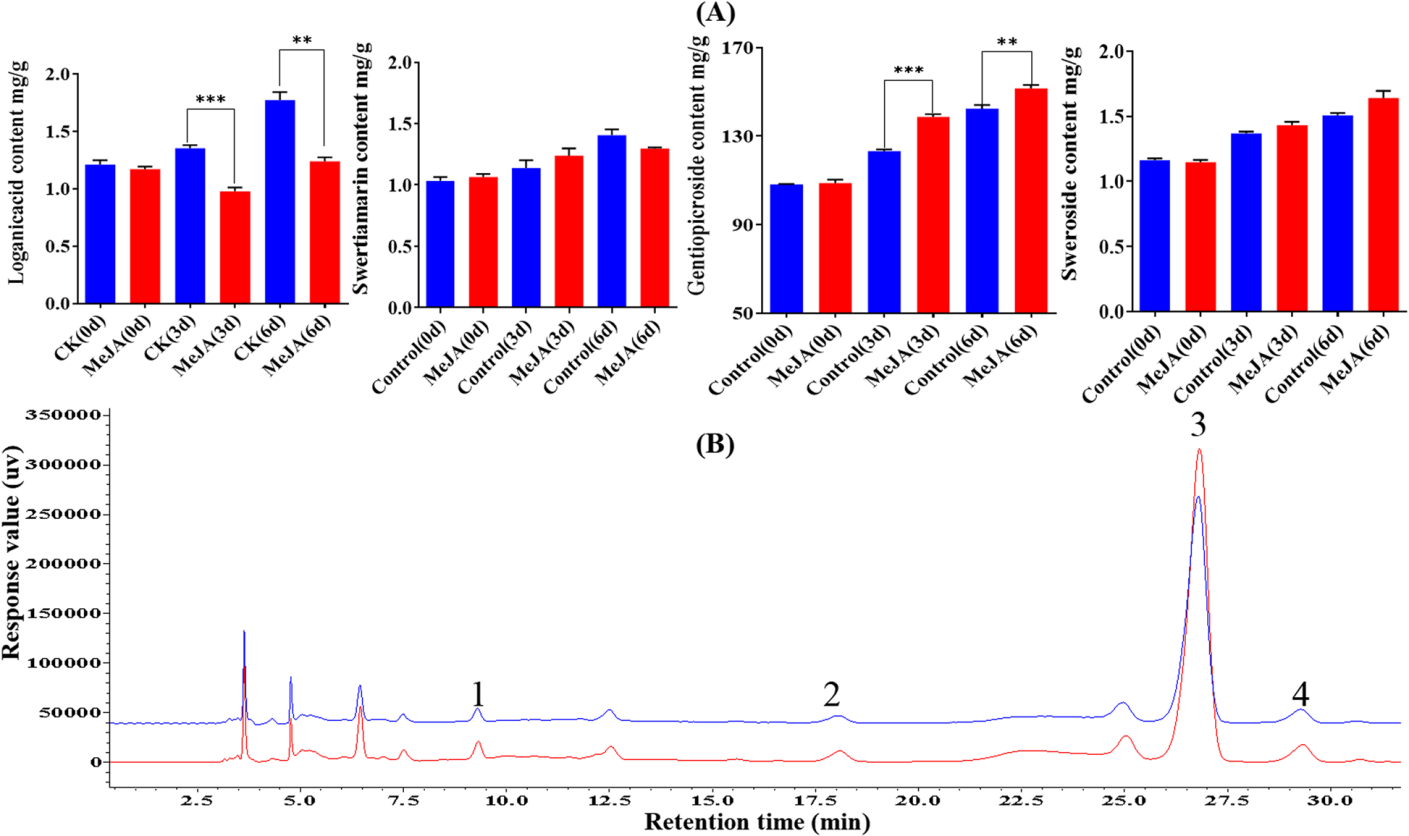
(A) The determination of the content of the four compounds (loganic acid, sweroside, gentiopicroside and swertiamarin) in the seedlings of *G. macrophylla* treated with MeJA. The red columns represent the content determination results of *G. macrophylla* seedlings treated with MeJA. The blue columns represent the content determination results of *G. macrophylla* seedlings without MeJA treatment (Control). The asterisks (*) represent significant differences (*, *P* < 0.05; **, *P* < 0.01; ***, *P* < 0.001; and ****, *P* < 0.0001). (B) Chromatograms for the determination of the content of four compounds in *G. macrophylla* seedlings for 3 days. The red chromatogram shows the content determination chromatogram of *G. macrophylla* seedlings treated with MeJA. The blue chromatogram shows the content determination of *G. macrophylla* seedlings without MeJA treatment (Control). 1: Loganic acid, 2: Swertiamarin, 3: Gentiopicroside, 4: Sweroside

**Fig. 9:**
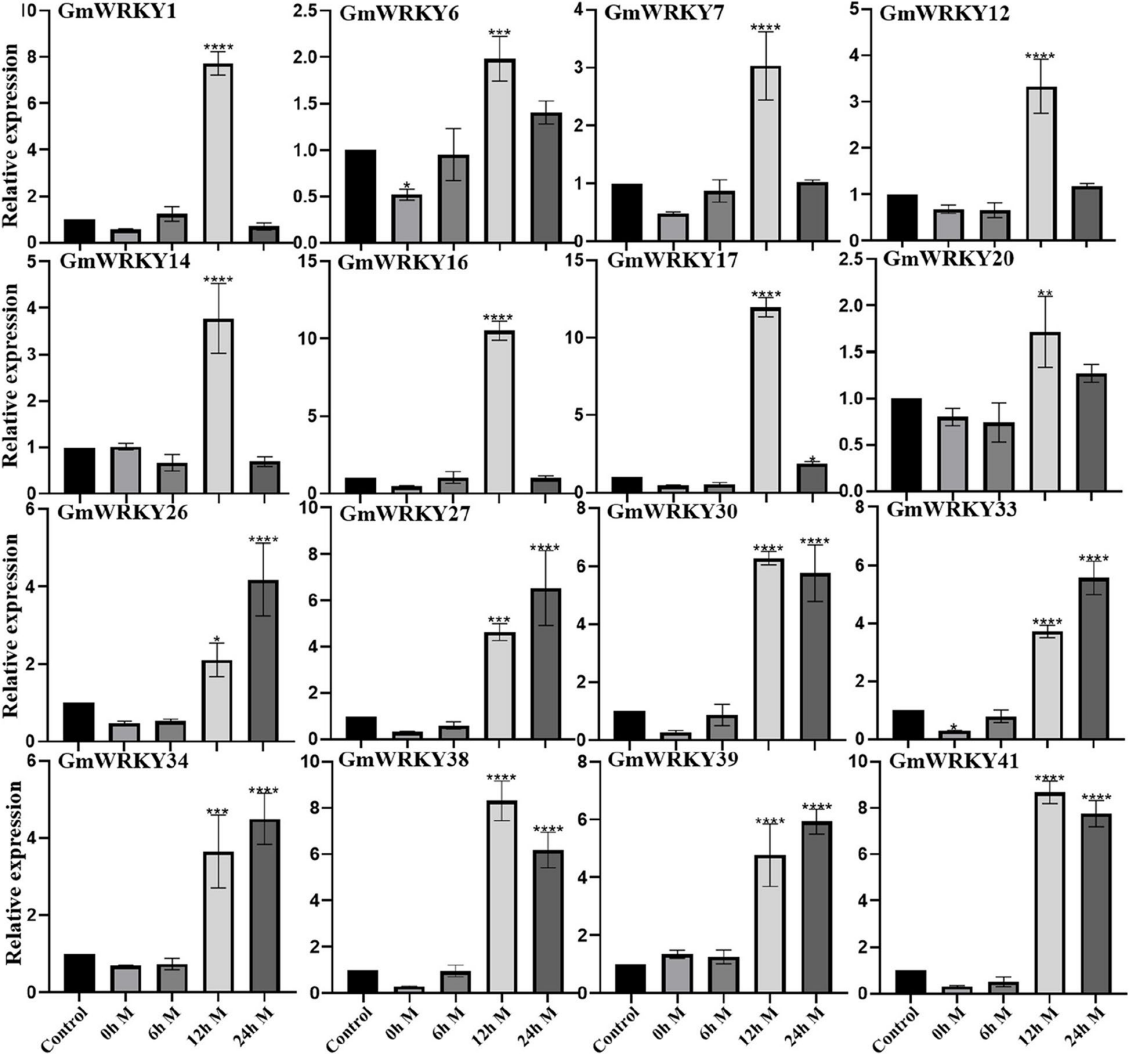
Analysis of *GmWRKY* expression in MeJA-treated seedlings of *G. macrophylla* (0 h, 6 h, 12 h and 24 h) tested by RT–qPCR. The CK (the seedling of *G. macrophylla* not treated with MeJA) was used as the blank control. The asterisks (*) represent significant differences (*, *P* < 0.05; **, *P* < 0.01; ***, *P* < 0.001; and ****, *P* < 0.0001)

**Fig. 10:**
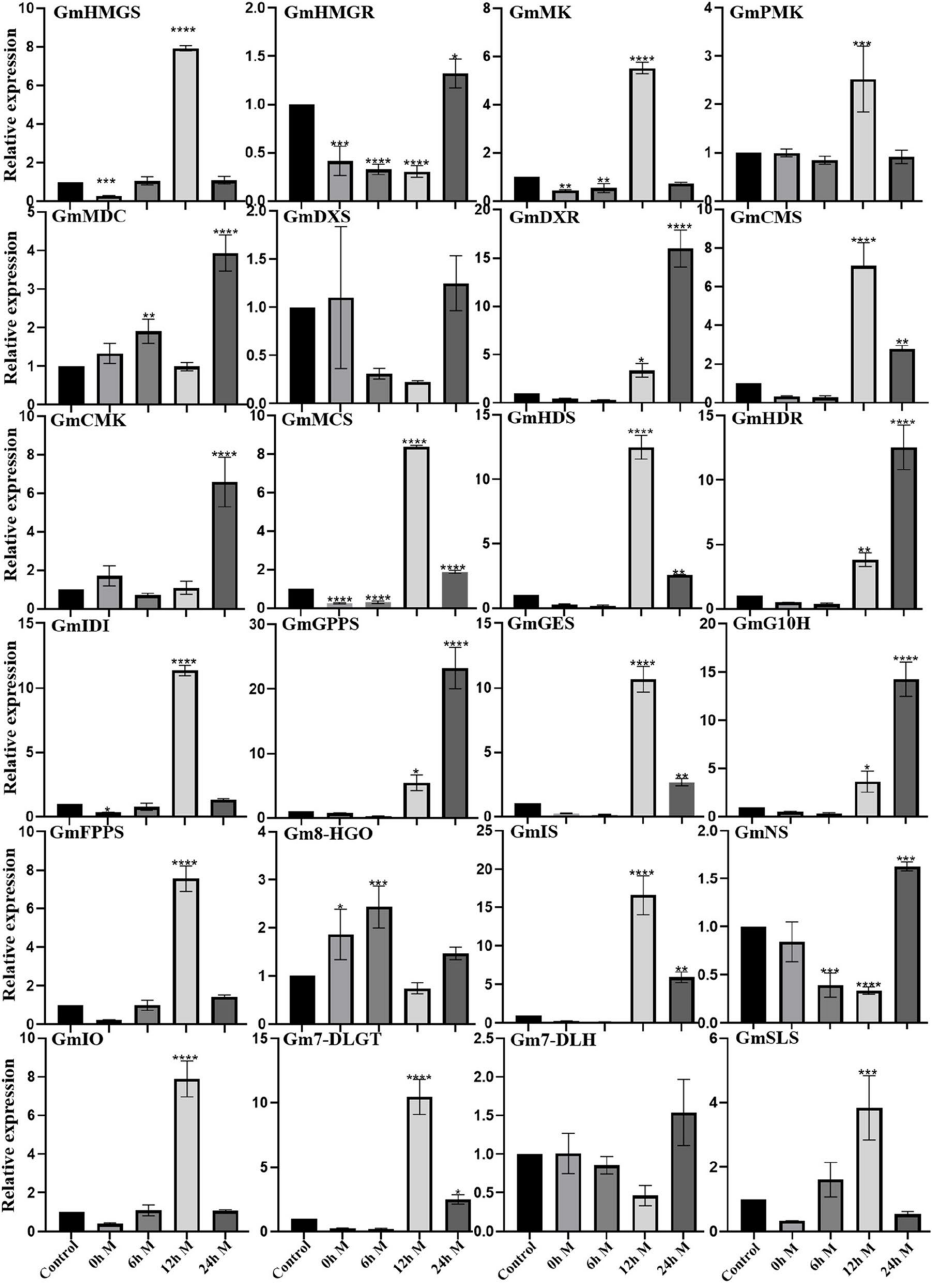
Analysis of the gene expression involved in secoiridoid biosynthesis in MeJA-treated seedlings of *G. macrophylla* (0 h, 6 h, 12 h and 24 h) tested by RT–qPCR. The CK (the seedling of *G. macrophylla* not treated with MeJA) was used as the blank control. The asterisks (*) represent significant differences (*, *P* < 0.05; **, *P* < 0.01; ***, *P* < 0.001; and ****, *P* < 0.0001)

Under the cognition that MeJA treatment could induce the expression of *GmWRKYs* in a previous study, to further verify the above experimental results that *GmWRKYs* were involved in the regulation of secoiridoid biosynthesis, seedlings of *G. macrophylla* treated with MeJA at the four-leaf stage were cultivated and collected; thus*, GmWRKY* expression, enzyme-encoding gene expression, and the content of the components in them were analysed. The results of the content determination (Fig. 8, Additional file 4: Fig. S4 and Additional file 14: Table S10) showed that the gentiopicroside content was significantly increased, and the sweroside content was gently increased, while the swertiamarin content showed no great change, whereas the loganic acid content was sharply decreased. The results of the RT–qPCR (Fig. 9 and Fig. 10) showed that the expression levels of 16 *GmWRKYs* were upregulated by MeJA treatment, of which 11 (*GmWRKY1*, *6*, *7*, *12*, *14*, *16*, 17, *20*, *30*, *38* and *41*) presented significant upregulation at 12 h but downregulation at 24 h; 5 (*GmWRKY26*, *27 33 34 39*) presented significant upregulation at 24 h. Meanwhile, some genes involved in secoiridoid biosynthesis shared a similar expression pattern with *GmWRKYs*, such as the genes *GmHMGS*, *GmMK* and *GmPMK* in the MVA pathway, the genes *GmCMS*, *GmMCS* and *GmHDS* in the MEP pathway, and the genes *GmIDI*, *GmGES*, *GmFPPS*, *GmIS*, *GmIO*, *Gm7-DLGT* and *GmSLS* in the characteristic secoiridoid pathway, presenting significant upregulation at 12 h but downregulation at 24 h; and 6 genes (*GmDXR*, *GmCMK*, *GmHDR*, *GmGGPS*, *GmG10H*, *GmNS*) involved in secoiridoid biosynthesis presented significant upregulation at 24 h, suggesting the same regulatory mechanism by MeJA between the *GmWRKYs* and the pathway biosynthetic genes.

## Discussion

*G. macrophylla*, the flowers of which are used as Tibetan medicine [41], while the roots of which are used as traditional Chinese medicine, is widely used in the pharmaceutical industry. Secoiridoids, as the main bioactive components, exhibit many therapeutic activities [42], with swertiamarin and gentiopicroside being regarded as two promising new natural drugs [43, 44]. Bioengineering studies to increase yields of swertiamarin [45] or gentiopicroside [46] have been carried out in recent years. Transcription factors are powerful tools to regulate the expression of synthetic pathway genes and improve the yield and quality of bioactive compounds [47]. Due to rare research on *G. macrophylla*, WRKY transcription factors in the regulation of secoiridoid biosynthesis were studied in this paper.

Herein, a total of 42 *GmWRKYs* were obtained from the *G. macrophylla* genome by bioinformatics analysis and divided into 3 groups (12 in Group I, 25 in Group II, and 5 in Group III). Obviously, group II was the largest group in *G. macrophylla*, which was similar to the results of *Panax ginseng* [28], *Eucommia ulmoides* [48], *Taraxacum kok-saghyz* [49] and *Bupleurum chinense* [50]. Considering that the distribution discrepancy of the groups implied different evolutionary processes in plant development [48, 51], the above data indicated that *GmWRKYs* in group II underwent more gene duplications during the process of evolution. Moreover, the typical characteristics of the WRKYs were conserved in structure but versatile in function. Some genes, such as *ApWRKYs* (*Andrographis paniculata*) [52] or *GmWRKY* (Soybean) [53] with similar protein sequence structures, showed similar expression patterns and performed similar biological functions, while others, such as *MnWRKY* (Mulberry) [54], *SiWRKY* (Sesame) [55] and *GmWRKYs* (*Gentiana macrophylla*), in the same group or subgroup shared highly similar motif compositions and gene structures but different expression patterns and functions.

In addition to *AtWRKYs* in *A. thaliana* [56] and *TkWRKYs* in *Taraxacum kok-saghyz* [49], most *GmWRKYs* contained a WRKY domain prominent feature composed of 60 amino acids with a conserved WRKYGQK heptapeptide sequence at the N-terminus. In most cases, the WRKY domain was very conserved; however, variant motifs such as WRKYGEK, WRKYGKK, WRICGQK, WSKYEQK and WRKYSEK were also found [57]. *GmWRKY9* sequence variations occurred and were replaced by the WRKYGKK motif. As reported that the variants might change the DNA binding specificities in the interactions of *WRKY* genes with downstream target genes [58, 59], the nature of *GmWRKY9* needs to be further explored in the future.

As shown in *Catharanthus roseus* [22] and *Salvia miltiorrhiza* [27], WRKY transcription factors always play important regulatory roles in the synthesis of secondary metabolites, while the expression patterns of enzyme-encoding genes involved in the formation of secondary metabolites in plants were significantly related to the distribution of metabolites. Thus, correlation analysis was conducted on the expression of *GmWRKYs*, the enzyme-encoding genes in the secoiridoid biosynthesis pathway, and the contents of loganinic acid, swertiamarin, gentiopicroside, and sweroside. As shown in Fig. 6, 32 *GmWRKYs* were significantly correlated with 23 enzymes. As shown in Figs. 7B and 8 *GmWRKYs* were positively correlated with gentiopicroside, while 4, 3 and 8 *GmWRKYs* were negatively correlated with loganic acid, swertiamarin and sweroside, respectively. In addition, the mechanism for *GmWRKYs* to regulate these metabolites was determined as the WRKY sequence initially binding to W-box elements [60], observed in the promoters of metabolic pathway enzyme-encoding genes, interacting with the target genes, and finally affecting the accumulation of secondary metabolites in *G. macrophylla.*

*AaGSW1* could be directly regulated by the jasmonic acid (JA) positive regulator *AaMYC2* in *Artemisia annua*, and overexpression of *AaGSW1* significantly increased the content of artemisinin and dihydroartemisinin [61]; *TcWRKY1* increased after JA induction in suspension cells of *Taxus chinensis*, and overexpression of *TcWRKY1* increased the expression of the *DBAT* gene, while RNA interference reduced the transcription level of the *DBAT* gene, a key rate-limiting enzyme in the Taxol biosynthesis pathway [62]. These studies revealed that WRKY transcription factors were involved in the response to JA signals, thus directly or indirectly affecting the accumulation of various plant secondary metabolites. Because *GmWRKYs* had hormone MeJA responsive elements in the promoter region, *G. macrophylla* seedlings were treated with MeJA. However, studies have not reached a consensus on the selection of time points for studying the correlation between genes and metabolites. The main reason is that the regulatory relationship between metabolites and genes is complex. The response of gene expression to exogenous inducer treatment occurs earlier than the accumulation of secondary metabolites; however, the front-to-back effect between them needs to consider their different rates and durations of changes in different plants or in the same plants with different test objectives; thus, measuring gene expression and metabolite content at different time points can better capture their correlation. For *G. macrophylla*, after MeJA treatment, changes in gene expression appeared within 24 h, but the contents of the metabolites changed from 3 to 6 days. Thus, they were chosen as the time points for this study. As shown in Fig. 8, the content of gentiopicroside was significantly increased after MeJA treatment, while the loganic acid content was sharply decreased, whereas sweroside and swertiamarin content showed no significant change. As shown in Figs. 9 and 10, the expression of 16 *GmWRKYs* and 22 enzyme-encoding genes were significantly increased after MeJA treatment, in which the expression of 8 *GmWRKYs* and 13 enzymes were significantly increased as well as the content of gentiopicroside, verifying the positive correlation indicated in the results of different tissues, while the expression of *4 GmWRKYs* and 14 enzymes were significantly increased in contrast to the reduction of loganic acid content, verifying the negative correlation indicated in different tissues. In addition, after treatment with MeJA, the expression of *GmWRKYs* and the enzyme-encoding genes related to swertiamarin and sweroside were also increased; however, their contents showed no significant change. Thus, 8 *GmWRKYs* (*GmWRKY1*, *6*, *12*, *17*, *33*, *34*, *38* and *39*) regulating the synthesis of gentiopicroside and 4 *GmWRKYs* (*GmWRKY7*, *14*, *26* and *41*) regulating the synthesis of loganic acid were identified in *G. macrophylla.* These results indicated that WRKY transcription factors were most likely involved in secoiridoid biosynthesis in *G. macrophylla*. This study provides a theoretical basis for further exploring the mechanism of WRKY transcription factors in the regulation of secondary metabolites in *G. macrophylla.*

## Conclusion

**Fig. 11:**
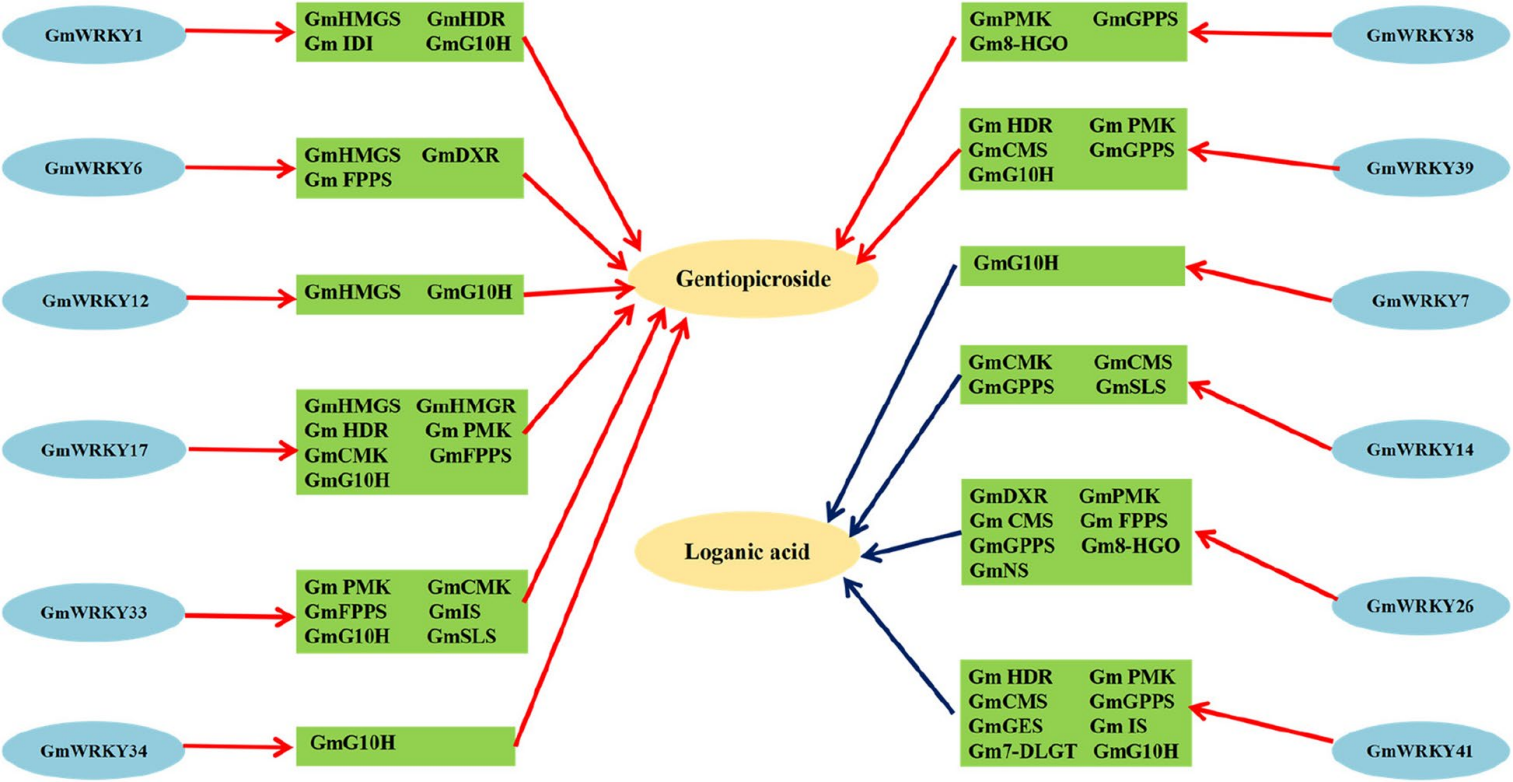
Predicted network diagram of GmWRKY pathway genes and secondary metabolites. The yellow ellipse presents the secondary metabolites, the light blue ellipse presents the *GmWRKYs*, and the green rectangle presents the pathway genes. The red arrow indicates positive regulation, and the blue arrow indicates negative regulation

In this study, 42 *WRKY* genes were identified in the *G. macrophylla* genome and divided into 3 groups (12 in Group I, 25 in Group IIa-e, and 5 in Group III) by sequence alignment, phylogenetic analysis, gene structure analysis and conserved motif analysis. Based on the study of the relationship of *WRKY* genes with the enzyme-encoding genes in the secondary metabolism pathway and the secondary metabolites and the experiment in which seedlings were treated with MeJA, 8 *GmWRKYs* (*GmWRKY1*, *6*, *12*, *17*, *33*, *34*, *38* and *39*) regulating the synthesis of gentiopicroside and 4 *GmWRKYs* (*GmWRKY7*, *14*, *26* and *41*) regulating the synthesis of loganic acid were found (Fig. 11). Our study has generated an important resource for further studies of WRKY transcription factors in *G. macrophylla* and provided a clue for further investigating *WRKY* gene function in secondary metabolite accumulation.

### Supplementary Information

**Additional file 1: Figure S1.** Multiple sequences alignment of the conserved domain of WRKY transcription factors in *G. macrophylla*.

**Additional file 2: Figure S2.** Phylogenetic tree of WRKYs from *G. macrophylla* and *A. thaliana*.

**Additional file 3: Figure S3.** The gene structure and the distribution of conserved motifs within each *GmWRKYs* in *G. macrophylla*.

**Additional file 4: Figure S4.** Chromatograms for the determination of four kinds of standards.

**Additional file 5: Table S1.** Identification and protein characterization of the *GmWRKY* genes.

**Additional file 6: Table S2.** Conserved motifs identified in WRKY transcription factors from *G. macrophylla* by MEME analysis.

**Additional file 7: Table S3.** Gene locus number of *GmWRKYs* across the chromosomes of *G. macrophylla.*

**Additional file 8: Table S4.** Tandem duplication events have been observed in the 42GmWRKY genes.

**Additional file 9: Table S5.** Enzyme genes involved in the biosynthesis of secoiridoids.

**Additional file 10: Table S6.** Correlation analysis results of *GmWRKYs* and the enzyme genes involved in secoiridoids biosynthesis.

**Additional file 11: Table S7.** Regulatory elements targeted by WRKY transcription factors in the promoters of the enzyme-coding genes of the secoiridoids biosynthesis pathway.

**Additional file 12: Table S8.** The contents of four representative components (loganic acid, swertiamarin, gentiopicroside and swerosides) in different tissues.

**Additional file 13: Table S9.** Primer sequences of the candidate WRKYs and enzyme genes used for RT-qPCR analyses.

**Additional file 14: Table S10.** The contents of the four compounds (loganic acid, swertiamarin gentiopicroside and sweroside) in *G. macrophylla* seedlings treated by MeJA.

## Data Availability

All data included in this study are available upon request by contact with the corresponding author.

## References

[1] Lu YY, Yang YM, Ma XH, Zhang XB, Zhu SD, Jin L (2016). Ecology suitability study of Chinese materia medica *Gentianae Macrophyllae* Radix. China J Chin Materia Med.

[2] Wang YM, Xu M, Wang D, Yang CR, Zeng Y, Zhang YJ (2013). Anti-inflammatory compounds of “Qin-Jiao”, the roots of *Gentiana dahurica* (Gentianaceae). J Ethnopharmacol.

[3] Yu F, Yu F, Li R, Wang R (2004). Inhibitory effects of the *Gentiana macrophylla* (Gentianaceae) extract on rheumatoid arthritis of rats. J Ethnopharmacol.

[4] Jia N, Chu W, Li Y, Ding L, Duan J, Cui J, Cao S, Zhao C, Wu Y, Wen A (2016). Iridoid glycosides from the flowers of Gentiana macrophylla Pall. ameliorate collagen-induced arthritis in rats. Journal of Ethnopharmacology.

[5] Wang Y, Ahmad B, Duan B, Zeng R, Huang L (2016). Chemical and genetic comparative analysis of *Gentiana crassicaulis* and *Gentiana macrophylla*. Chem Biodivers.

[6] Kou Y, Yi X, Li Z, Ai Y, Ma S, Chen Q (2022). A comparative transcriptomic with UPLC-Q-Exactive MS reveals differences in gene expression and components of iridoid biosynthesis in various parts of *Gentiana macrophylla*. Genes..

[7] Liao P, Hemmerlin A, Bach TJ, Chye M-L (2016). The potential of the mevalonate pathway for enhanced isoprenoid production. Biotechnol Adv.

[8] Zeng L, Dehesh K (2021). The eukaryotic MEP-pathway genes are evolutionarily conserved and originated from Chlaymidia and cyanobacteria. BMC Genomics.

[9] Patra B, Schluttenhofer C, Wu Y, Pattanaik S, Yuan L (2013). Transcriptional regulation of secondary metabolite biosynthesis in plants. Gene Regul Mechan.

[10] Yang CQ, Fang X, Wu XM, Mao YB, Wang LJ, Chen XY (2012). Transcriptional regulation of plant secondary metabolism. J Integr Plant Biol.

[11] Rushton PJ, Somssich IE, Ringler P, Shen QJ (2010). WRKY transcription factors. Trends Plant Sci.

[12] Grzechowiak M, Ruszkowska A, Sliwiak J, Urbanowicz A, Jaskolski M, Ruszkowski M (2022). New aspects of DNA recognition by group II WRKY transcription factor revealed by structural and functional study of AtWRKY18 DNA binding domain. Int J Biol Macromol.

[13] Jing C, Wang D, Liu Z, Chen X, Dong H, Wu X (2023). Identification of the WRKY gene family in apricot and its response to drought stress. Hortic Environ Biotechnol.

[14] Du P, Wu Q, Liu Y, Cao X, Yi W, Jiao T, Hu M, Huang Y (2022). WRKY transcription factor family in lettuce plant (*Lactuca sativa*): Genome-wide characterization, chromosome location, phylogeny structures, and expression patterns. PeerJ.

[15] Eulgem T, Rushton PJ, Robatzek S, Somssich IE (2000). The WRKY superfamily of plant transcription factors. Trends in PlantScience.

[16] Ulker B, Somssich IE (2004). WRKY transcription factors: from DNA binding towards biological function. Curr Opin Plant Biol.

[17] Chen L, Song Y, Li S, Zhang L, Zou C, Yu D (2012). The role of WRKY transcription factors in plant abiotic stresses. Biochem Biophys Acta.

[18] Liang W, Yang B, Yu B-J, Zhou Z, Li C, Jia M, Sun Y, Zhang Y, Wu F, Zhang H (2013). Identification and analysis of MKK and MPK gene families in canola (Brassica napus L.). BMC Genom.

[19] Yang B, Jiang Y, Rahman MH, Deyholos MK, Kav NNV (2009). Identification and expression analysis of WRKY transcription factor genes in canola (Brassica napus L.) in response to fungal pathogens and hormone treatments. BMC Plant Biol.

[20] Li W, Pang S, Lu Z, Jin B (2020). Function and mechanism of WRKY transcription factors in abiotic stress responses of plants. Plants.

[21] Fu X, Peng B, Hassani D, Xie L, Liu H, Li Y, Chen T, Liu P, Tang Y, Li L (2021). AaWRKY9 contributes to light- and jasmonate-mediated to regulate the biosynthesis of artemisinin in *Artemisia annua*. New Phytol.

[22] Suttipanta N, Pattanaik S, Kulshrestha M, Patra B, Singh SK, Yuan L (2011). The transcription factor CrWRKY1 positively regulates the terpenoid indole alkaloid biosynthesis in *Catharanthus roseus*. Plant Physiol.

[23] Chen Y, Zhang H, Zhang M, Zhang W, Ou Z, Peng Z, Fu C, Zhao C, Yu L (2021). Salicylic acid-responsive factor TcWRKY33 positively regulates taxol biosynthesis in *Taxus chinensis* in direct and indirect ways. Front Plant Sci.

[24] Li X, He L, An X, Yu K, Meng N, Duan C, Pan Q-H (2020). VviWRKY40, a WRKY transcription factor, regulates glycosylated monoterpenoid production by VviGT14 in grape berry. Genes..

[25] Ishiguro S, Nakamura K (1994). Characterization of a cDNA encoding a novel DNA-binding protein, SPF1, that recognizes SP8 sequences in the 5' upstream regions of genes coding for sporamin and beta-amylase from sweet potato. Mol Gen Genet.

[26] Yamada Y, Nishida S, Shitan N, Sato F (2021). Genome-Wide Profiling of WRKY Genes Involved in Benzylisoquinoline Alkaloid Biosynthesis in California Poppy (*Eschscholzia californica*). Front Plant Sci.

[27] Yu H, Guo W, Yang D, Hou Z, Liang Z (2018). Transcriptional profiles of SmWRKY family genes and their putative roles in the biosynthesis of tanshinone and phenolic acids in *Salvia miltiorrhiza*. Int J Mol Sci..

[28] Di P, Wang P, Yan M, Han P, Huang X, Yin L, Yan Y, Xu Y, Wang Y (2021). Genome-wide characterization and analysis of WRKY transcription factors in *Panax ginseng*. BMC Genomics.

[29] Wei H, Chen S, Niyitanga S, Liu T, Qi J, Zhang L (2022). Genome-wide identification and expression analysis response to GA3 stresses of WRKY gene family in seed hemp (*Cannabis sativa* L). Gene.

[30] Cao X, Guo X, Yang X, Wang H, Hua W, He Y, Kang J, Wang Z (2016). Transcriptional responses and gentiopicroside biosynthesis in methyl Jasmonate-treated *Gentiana macrophylla* seedlings. PLoS ONE.

[31] Duvaud S, Gabella C, Lisacek F, Stockinger H, Ioannidis V, Durinx C (2021). Expasy, the Swiss bioinformatics resource portal, as designed by its users. Nucleic Acids Res.

[32] Chou KC, Shen HB (2010). Plant-mPLoc: a top-down strategy to augment the power for predicting plant protein subcellular localization. PLoS ONE.

[33] Kumar S, Stecher G, Tamura K (2016). MEGA7: molecular evolutionary genetics analysis version 7.0 for bigger datasets. Mol Biol Evol.

[34] Chen C, Chen H, Zhang Y, Thomas HR, Frank MH, He Y, Xia R (2020). TBtools: an integrative toolkit developed for interactive analyses of big biological data. Mol Plant.

[35] Bailey TL, Boden M, Buske FA, Frith M, Grant CE, Clementi L, Ren J, Li WW, Noble WS (2009). MEME SUITE: tools for motif discovery and searching. Nucleic Acids Res.

[36] Lescot M, Déhais P, Thijs G, Marchal K, Moreau Y, Van de Peer Y, Rouzé P, Rombauts S (2002). PlantCARE, a database of plant cis-acting regulatory elements and a portal to tools for in silico analysis of promoter sequences. Nucleic Acids Res.

[37] Minoru K, Miho F, Yoko S, Masayuki K, Mari I-W (2023). KEGG for taxonomy-based analysis of pathways and genomes. Nucleic Acids Res.

[38] Zhou T, Bai G, Hu Y, Ruhsam M, Yang Y, Zhao Y (2022). De novo genome assembly of the medicinal plant *Gentiana macrophylla* provides insights into the genomic evolution and biosynthesis of iridoids. DNA Res.

[39] Yihan H, Hailing Y, Wenping H, Yaya H, Zhezhi W (2016). Selection and validation of reference genes for quantitative real-time PCR in *Gentiana macrophylla*. Front Plant Sci.

[40] Holub EB (2001). The arms race is ancient history in Arabidopsis, the wildflower. Nat Rev Genet.

[41] Zhao L, Ye J, Wu G-T, Peng X-J, Xia P-F, Ren Y (2015). Gentiopicroside prevents interleukin-1 beta induced inflammation response in rat articular chondrocyte. J Ethnopharmacol.

[42] Dinda B, Debnath S, Harigaya Y (2007). Naturally occurring secoiridoids and bioactivity of naturally occurring iridoids and secoiridoids. A review, part 2. Cheminform.

[43] Muhamad Fadzil NS, Sekar M, Gan SH, Bonam SR, Wu YS, Vaijanathappa J, Ravi S, Lum PT, Dhadde SB (2021). Chemistry, pharmacology and therapeutic potential of Swertiamarin - a promising natural lead for new drug discovery and development. Drug Des Dev Ther.

[44] Wu S, Ning Y, Zhao Y, Sun W, Thorimbert S, Dechoux L, Sollogoub M, Zhang Y (2017). Research progress of natural product Gentiopicroside - a Secoiridoid compound. Mini-Rev Med Chem.

[45] Wang J, Liu Y, Cai Y, Zhang F, Xia G, Xiang F (2010). Cloning and functional analysis of geraniol 10-hydroxylase, a cytochrome P450 from *Swertia mussotii* Franch. Biosci Biotechnol Biochem.

[46] Cai Y, Yanling L, Zhenhua Z, Feng X, Fengning X, Guangmin X (2009). High-frequency embryogenesis and regeneration of plants with high content of gentiopicroside from the Chinese medicinal plant *Gentiana straminea* Maxim. In Vitro Cell Dev Biol Plant: J Tissue Cult Assoc.

[47] Vom Endt D, Kijne JW, Memelink J (2002). Transcription factors controlling plant secondary metabolism: what regulates the regulators?. Phytochemistry.

[48] Liu J, Wang X, Chen Y, Liu Y, Wu Y, Ren S, Li L (2021). Identification, evolution and expression analysis of WRKY gene family in *Eucommia ulmoides*. Genomics.

[49] Cheng Y, Luo J, Li H, Wei F, Zhang Y, Jiang H, Peng X (2022). Identification of the WRKY gene family and characterization of stress-responsive genes in *Taraxacum kok*-*saghyz* Rodin. Int J Mol Sci.

[50] Wu SR, Gao K, Liu X, Xu J, Wei JH, Sui C (2017). Identification of WRKY transcription factors related to Saikosaponin biosynthesis in adventitious roots of *Bupleurum chinense*. Chin Herb Med.

[51] Ayadi M, Hanana M, Kharrat N, Merchaoui H, Marzoug RB, Lauvergeat V, Rebaï A, Mzid R (2016). The WRKY transcription factor family in citrus: valuable and useful candidate genes for citrus breeding. Appl Biochem Biotechnol.

[52] Zhang R, Chen Z, Zhang L, Yao W, Ji A (2021). Genomic characterization of WRKY transcription factors related to andrographolide biosynthesis in *Andrographis paniculata*. Front Genet.

[53] Kurt F, Filiz E (2020). Biological network analyses of WRKY transcription factor family in soybean (*Glycine max*) under low phosphorus treatment. J Crop Sci Biotechnol.

[54] Baranwal VK, Negi N, Khurana P (2016). Genome-wide identification and structural, functional and evolutionary analysis of WRKY components of mulberry. Sci Rep.

[55] Li D, Liu P, Yu J, Wang L, Dossa K, Zhang Y, Zhou R, Wei X, Zhang X (2017). Genome-wide analysis of WRKY gene family in the sesame genome and identification of the WRKY genes involved in responses to abiotic stresses. BMC Plant Biol.

[56] Yamasaki K (2012). Structural basis for sequence-specific DNA recognition by an Arabidopsis WRKY transcription factor. J Biol Chem.

[57] Sáenz-Mata J, Salazar-Badillo FB, Jiménez-Bremont JF (2014). Transcriptional regulation of Arabidopsis thaliana WRKY genes under interaction with beneficial fungus *Trichoderma atroviride*. Acta Physiol Plant.

[58] Van Verk MC, Pappaioannou D, Neeleman L, Bol JF, Linthorst HJM (2008). A novel WRKY transcription factor is required for induction of PR-1a gene expression by salicylic acid and bacterial elicitors. Plant Physiol.

[59] Zhou QY, Tian AG, Zou HF, Xie ZM, Lei G, Huang J, Wang CM, Wang HW, Zhang JS, Chen SY (2008). Soybean WRKY-type transcription factor genes, GmWRKY13, GmWRKY21, and GmWRKY54, confer differential tolerance to abiotic stresses in transgenic Arabidopsis plants. Plant Biotechnol J.

[60] Schluttenhofer C, Yuan L (2015). Regulation of specialized metabolism by WRKY transcription factors. Plant Physiol.

[61] Chen M, Yan T, Shen Q, Lu X, Tang K (2017). GLANDULAR TRICHOME-SPECIFIC WRKY 1 promotes artemisinin biosynthesis in *Artemisia annua*. New Phytol.

[62] Zhang M, Chen Y, Nie L, Jin X, Liao W, Zhao S, Fu C, Yu L (2018). Transcriptome-wide identification and screening of WRKY factors involved in the regulation of taxol biosynthesis in *Taxus chinensis*. Sci Rep.

