# Supplementary material for: Genomic characterization of WRKY transcription factors related to secoiridoid biosynthesis in Gentiana macrophylla
Source: BMC Plant Biol. 2024 Jan 23;24:66. doi: 10.1186/s12870-024-04727-z (PMC10804491; doi:10.1186/s12870-024-04727-z)
Supplement: Supplementary file 1 — Additional file 1: Figure S1. Multiple sequences alignment of the conserved domain of WRKY transcription factors in G. macrophylla. [file 12870_2024_4727_MOESM1_ESM.docx]

**
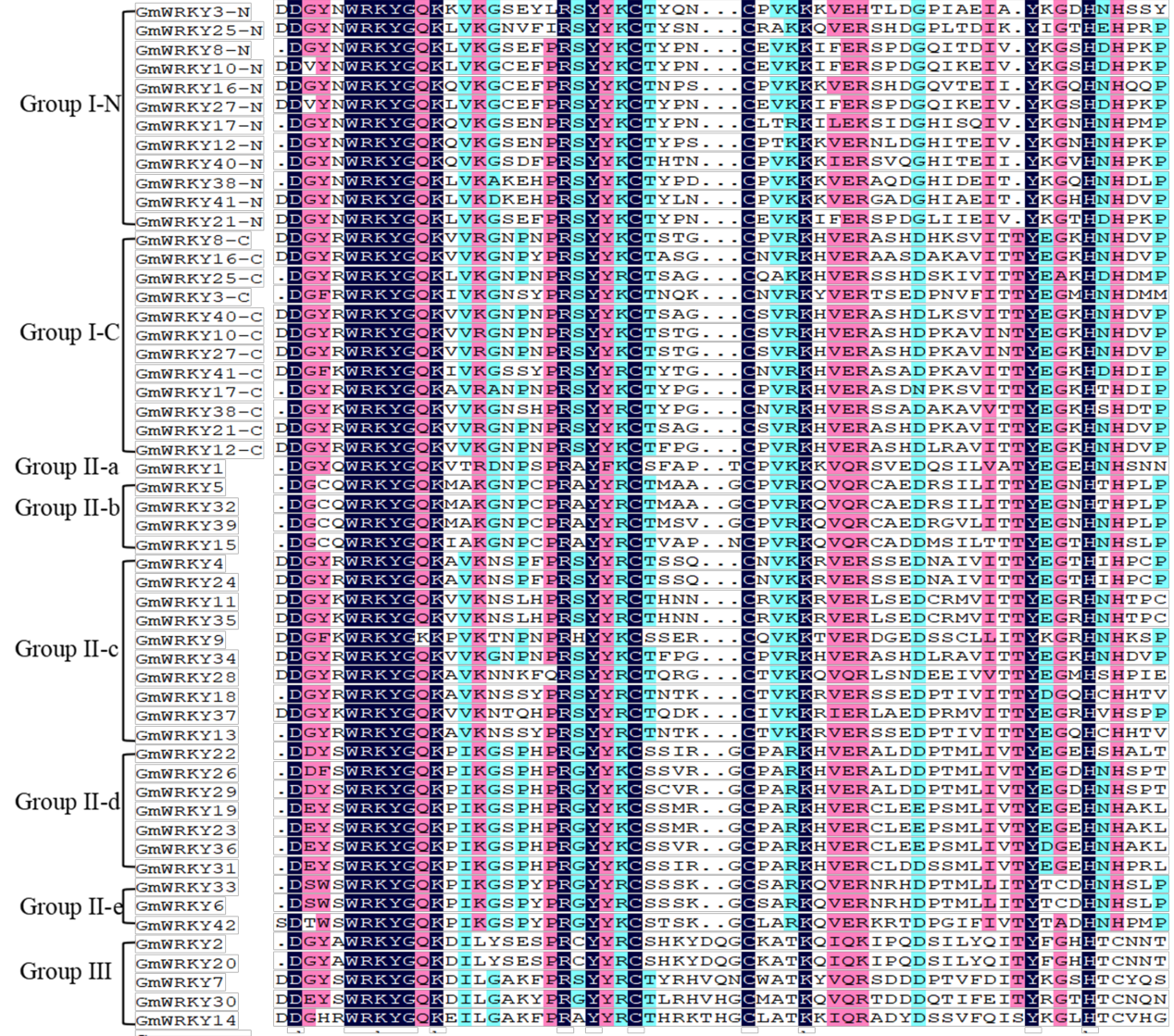
**

**Additional file 1: Figure S1** Multiple sequences alignment of the conserved domain of WRKY transcription factors in *G. macrophylla*. The WRKY domain of each group was blasted by the DNAMAN 9.0 software, and conserved amino acid residues were shown in dark blue.
