# Supplementary material for: Genomic characterization of WRKY transcription factors related to secoiridoid biosynthesis in Gentiana macrophylla
Source: BMC Plant Biol. 2024 Jan 23;24:66. doi: 10.1186/s12870-024-04727-z (PMC10804491; doi:10.1186/s12870-024-04727-z)
Supplement: Supplementary file 2 — Additional file 2: Figure S2. Phylogenetic tree of WRKYs from G. macrophylla and A. thaliana. [file 12870_2024_4727_MOESM2_ESM.docx]

**
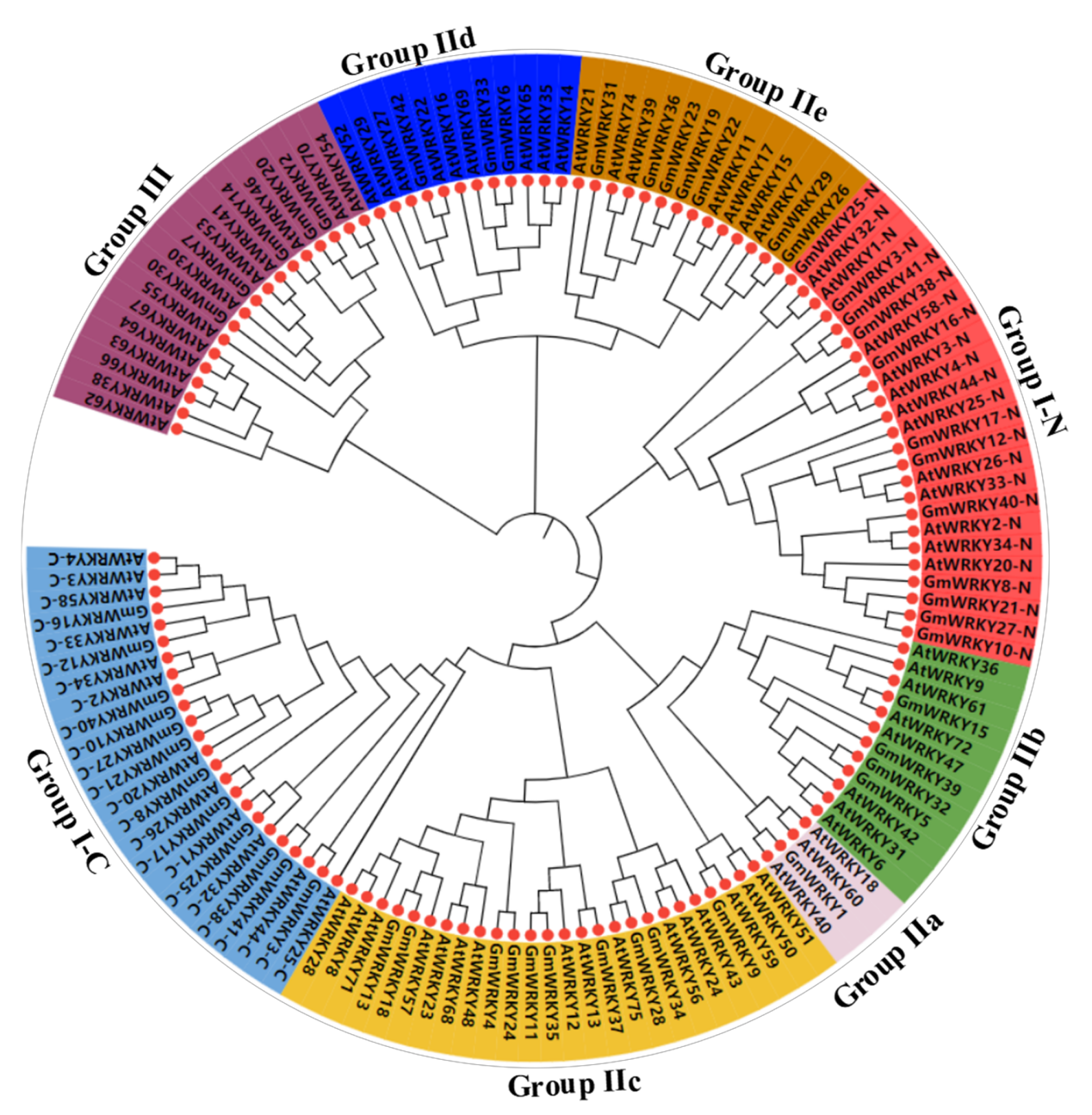
**

**Additional file 2: Figure S2** Phylogenetic tree of WRKYs from *G. macrophylla* and *A. thaliana*. Groups and subgroups are distinguished by different colors (Group I-N: red; Group I-C: light blue; Group IIa: pink; Group IIb: green; Group IIc: yellow; Group IId: dark blue; Group IIe: brown; Group III: purple).
