# Supplementary material for: Genomic characterization of WRKY transcription factors related to secoiridoid biosynthesis in Gentiana macrophylla
Source: BMC Plant Biol. 2024 Jan 23;24:66. doi: 10.1186/s12870-024-04727-z (PMC10804491; doi:10.1186/s12870-024-04727-z)
Supplement: Supplementary file 3 — Additional file 3: Figure S3. The gene structure and the distribution of conserved motifs within each GmWRKYs in G. macrophylla. [file 12870_2024_4727_MOESM3_ESM.docx]

**
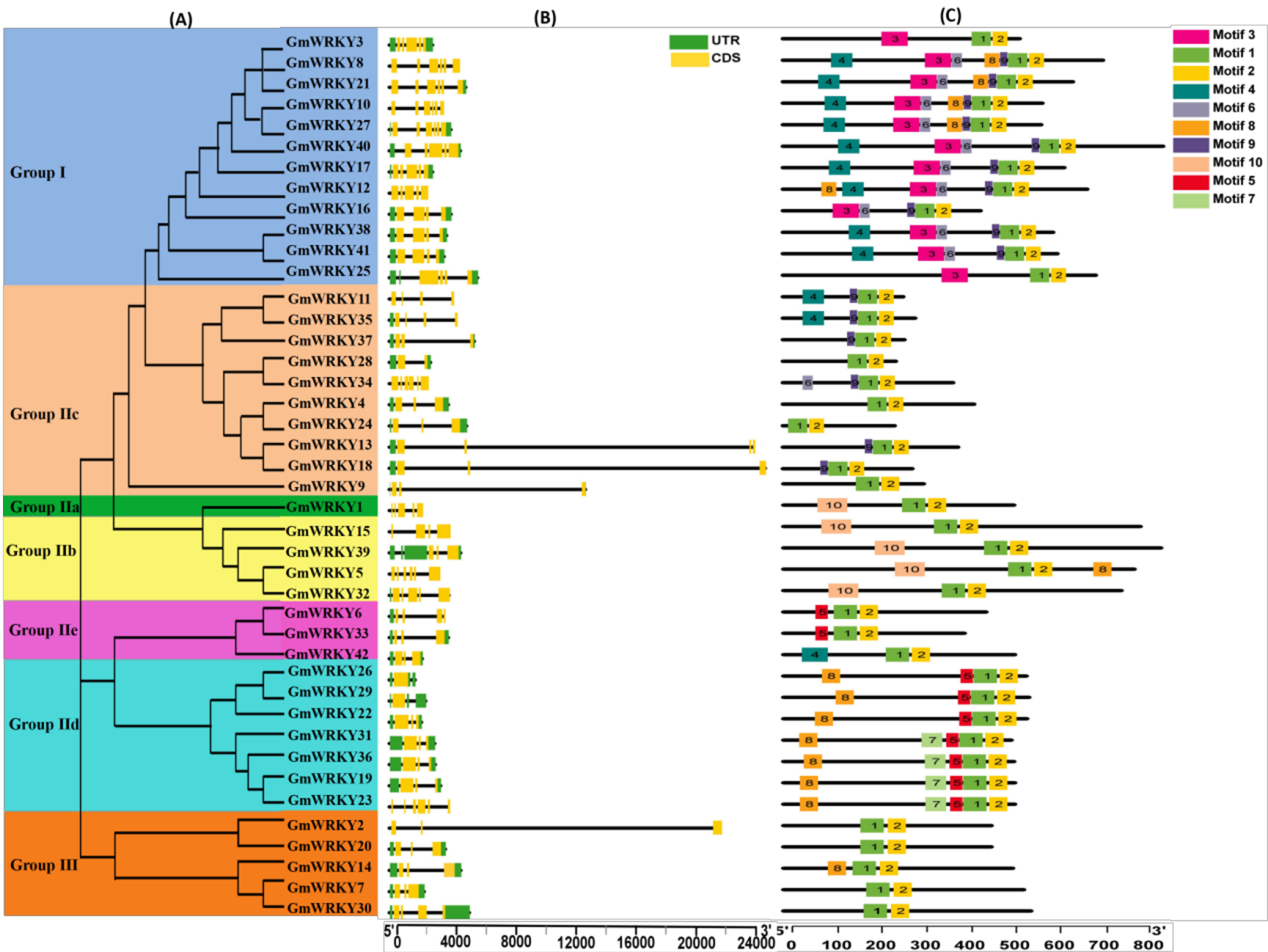
**

**Additional file 3: Figure S3** The gene structure and the distribution of conserved motifs within each *GmWRKYs* in *G. macrophylla*. (A) Phylogenetic tree of GmWRKYs proteins. (B)The gene structures of *GmWRKYs*. (C) The distribution of conserved motifs within each GmWRKYs protein.
