# Supplementary material for: Genomic characterization of WRKY transcription factors related to secoiridoid biosynthesis in Gentiana macrophylla
Source: BMC Plant Biol. 2024 Jan 23;24:66. doi: 10.1186/s12870-024-04727-z (PMC10804491; doi:10.1186/s12870-024-04727-z)
Supplement: Supplementary file 4 — Additional file 4: Figure S4. Chromatograms for the determination of four kinds of standards. [file 12870_2024_4727_MOESM4_ESM.docx]

**
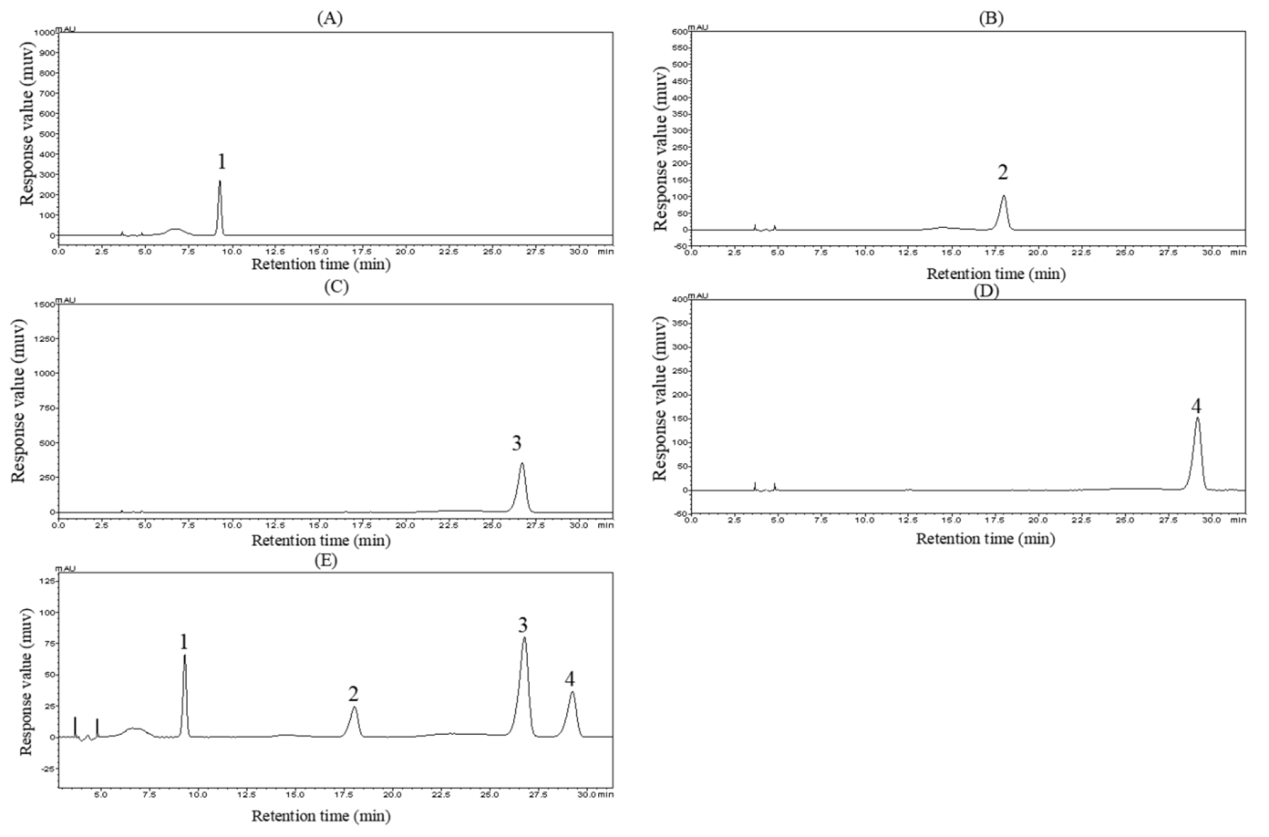
**

**Additional file 4: Figure S4** Chromatograms for the determination of four kinds of standards. (A) Chromatograms for the determination of loganic acid standards (Retention time: 9.289 min). (B) Chromatograms for the determination of swertiamarin standards (Retention time: 18.020 min). (C) Chromatograms for the determination of gentiopicroside standards (Retention time: 26.612 min). (D) Chromatograms for the determination of sweroside standards (Retention time: 29.225 min). (E) Chromatograms for the determination of loganic acid, swertiamarin, gentiopicroside and sweroside mixed of standards. 1: Loganic acid, 2: Swertiamarin, 3: Gentiopicroside, 4: Sweroside.
