# Supplementary material for: Genomic characterization of WRKY transcription factors related to secoiridoid biosynthesis in Gentiana macrophylla
Source: BMC Plant Biol. 2024 Jan 23;24:66. doi: 10.1186/s12870-024-04727-z (PMC10804491; doi:10.1186/s12870-024-04727-z)
Supplement: Supplementary file 12 — Additional file 12: Table S8. The contents of four representative components (loganic acid, swertiamarin, gentiopicroside and swerosides) in different tissues. [file 12870_2024_4727_MOESM12_ESM.docx]

**Additional file 12: Table S8** The contents of four representative components (loganic acid, swertiamarin, gentiopicroside and swerosides) in different tissues.

| **Tissues** | **Loganic acid (mg/g)** | **Swertiamarin**  **(mg/g)** | **Gentiopicroside (mg/g)** | **Sweroside**  **(mg/g)** |
| --- | --- | --- | --- | --- |
| Roots | 11.83 ± 0.52 | 4.13 ± 0.35 | 217.98±2.87 | 0.92 ± 0.07 |
| Stems | 12.24 ± 0.12 | 1.59 ± 0.05 | 50.82 ± 1.67 | 0.44 ± 0.02 |
| Leaves | 3.01 ± 0.18 | 2.02 ± 0.10 | 84.62 ± 1.54 | 0.22 ± 0.02 |
| Flowers | 7.87 ± 0.18 | 3.07 ± 0.31 | 39.67 ± 0.67 | 2.22 ± 0.01 |
| Seeds | 20.69 ± 0.63 | 5.13 ± 0.10 | 13.62 ± 0.80 | 2.58 ± 0.06 |
