# Supplementary material for: Genomic characterization of WRKY transcription factors related to secoiridoid biosynthesis in Gentiana macrophylla
Source: BMC Plant Biol. 2024 Jan 23;24:66. doi: 10.1186/s12870-024-04727-z (PMC10804491; doi:10.1186/s12870-024-04727-z)
Supplement: Supplementary file 14 — Additional file 14: Table S10. The contents of the four compounds (loganic acid, swertiamarin gentiopicroside and sweroside) in G. macrophylla seedlings treated by MeJA. [file 12870_2024_4727_MOESM14_ESM.docx]

**Additional file 14: Table S10** The contents of the four compounds (loganic acid, swertiamarin gentiopicroside and sweroside) in *G. macrophylla* seedlings treated by MeJA.

| **Samples** | **Loganic acid (mg/g)** | **Swertiamarin**  **(mg/g)** | **Gentiopicroside (mg/g)** | **Sweroside (mg/g)** |
| --- | --- | --- | --- | --- |
| Control (0d) | 1.21 ± 0.05 | 1.03 ± 0.04 | 108.11 ± 0.37 | 1.16 ± 0.02 |
| MeJA (0d) | 1.17 ± 0.03 | 1.06 ± 0.04 | 108.783 ± 2.22 | 1.15 ± 0.05 |
| Control (3d) | 1.35 ± 0.04 | 1.14 ± 0.09 | 123.22 ± 1.12 | 1.37 ± 0.02 |
| MeJA (3d) | 0.98 ± 0.01 | 1.24 ± 0.09 | 138.70 ± 1.67 | 1.43 ± 0.03 |
| Control (6d) | 1.77 ± 0.10 | 1.40 ± 0.07 | 142.45 ± 2.34 | 1.51 ± 0.02 |
| MeJA (6d) | 1.24 ± 0.028 | 1.29 ± 0.01 | 151.56 ± 2.13 | 1.66 ± 0.04 |
